# The evolution of cardiolipin biosynthesis and maturation pathways and its implications for the evolution of eukaryotes

**DOI:** 10.1186/1471-2148-12-32

**Published:** 2012-03-13

**Authors:** Hai-Feng Tian, Jin-Mei Feng, Jian-Fan Wen

**Affiliations:** 1 State Key Laboratory of Genetic Resources and Evolution, Kunming Institute of Zoology, Chinese Academy of Sciences, Kunming, Yunnan Province 650223, China; 2 Graduate School of the Chinese Academy of Sciences, Beijing 100039, China

**Keywords:** Cardiolipin synthase, Phylogenetic distribution, Phylogenetic analysis, Mitochondrial endosymbiosis, Eukaryotic evolution

## Abstract

**Background:**

Cardiolipin (CL) is an important component in mitochondrial inner and bacterial membranes. Its appearance in these two biomembranes has been considered as evidence of the endosymbiotic origin of mitochondria. But CL was reported to be synthesized through two distinct enzymes--CLS_cap and CLS_pld in eukaryotes and bacteria. Therefore, how the CL biosynthesis pathway evolved is an interesting question.

**Results:**

Phylogenetic distribution investigation of CL synthase (CLS) showed: most bacteria have CLS_pld pathway, but in partial bacteria including proteobacteria and actinobacteria CLS_cap pathway has already appeared; in eukaryotes, Supergroup Opisthokonta and Archaeplastida, and Subgroup Stramenopiles, which all contain multicellular organisms, possess CLS_cap pathway, while Supergroup Amoebozoa and Excavata and Subgroup Alveolata, which all consist exclusively of unicellular eukaryotes, bear CLS_pld pathway; amitochondriate protists in any supergroups have neither. Phylogenetic analysis indicated the CLS_cap in eukaryotes have the closest relationship with those of alpha proteobacteria, while the CLS_pld in eukaryotes share a common ancestor but have no close correlation with those of any particular bacteria.

**Conclusions:**

The first eukaryote common ancestor (FECA) inherited the CLS_pld from its bacterial ancestor (e. g. the bacterial partner according to any of the hypotheses about eukaryote evolution); later, when the FECA evolved into the last eukaryote common ancestor (LECA), the endosymbiotic mitochondria (alpha proteobacteria) brought in CLS_cap, and then in some LECA individuals the CLS_cap substituted the CLS_pld, and these LECAs would evolve into the protist lineages from which multicellular eukaryotes could arise, while in the other LECAs the CLS_pld was retained and the CLS_cap was lost, and these LECAs would evolve into the protist lineages possessing CLS_pld. Besides, our work indicated CL maturation pathway arose after the emergence of eukaryotes probably through mechanisms such as duplication of other genes, and gene duplication and loss occurred frequently at different lineage levels, increasing the pathway diversity probably to fit the complicated cellular process in various cells. Our work also implies the classification putting Stramenopiles and Alveolata together to form Chromalveolata may be unreasonable; the absence of CL synthesis and maturation pathways in amitochondriate protists is most probably due to secondary loss.

## Accession number

The nucleotide sequences of the *Phaeodactylum tricornutum *CLS_cap identified by us have been submitted to GenBank and their accession numbers are JN088191 and JN088192.

## Background

Cardiolipin (CL) is an important phospholipid component of mitochondrial inner membrane and bacterial membrane. In mitochondria, CL stabilizes the respiratory complexes and the supercomplexes mainly made up of complex III/IV [1,2], and maintains the generation of ATP [3,4]; it is also involved in mitochondrial protein import, cell wall biogenesis, translational regulation, aging and apoptosis [2]. In bacteria, CL interacts with energy metabolism proteins such as succinate dehydrogenase [5], formate dehydrogenase-N [6], and respiratory complex [7], and is assembled into reaction centers [8,9], and is also involved in proper localization of proteins on membrane [10,11]. Whereas, no CL have ever been found in archaea yet [12].

**Figure 1:**
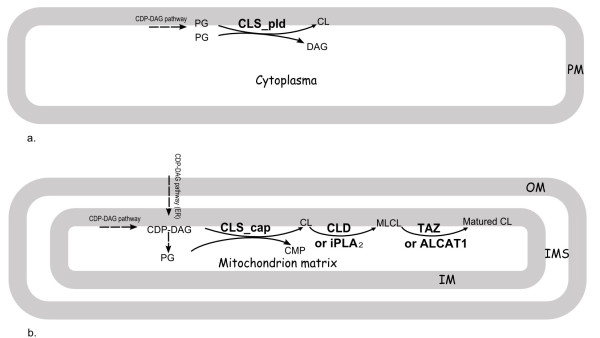
**Diagrams of two types of CL synthesis pathways occurring in bacteria and eukaryotic mitochondria, respectively**. (a) CLS_pld pathway in a bacterium; (b) CLS_cap and maturation pathways in a mitochondrion of a eukaryotic cell. ER, endoplasmic reticulum; PM, plasmamembrane; OM, out membrane of mitochondrion; IMS, intermembrane space; IM, innermembrane of mitochondrion. Dashed arrow indicates the upstream pathways that not displayed here.

CL is biosynthesized from two molecules of phosphatidylglycerols (PG) molecules in bacteria while from a PG and a Cytidine diphosphate diacylglycerol (CDP-DAG) in eukaryotes (Figure 1) [13]. In bacteria, the biosynthesis reaction is a reversible transesterification catalyzed by a kind of cardiolipin synthase (CLS) containing two phospholipase D (PLDc_2) domains--CLS_pld, while in eukaryotes, the reaction is not a reversible one catalyzed by another kind of CLS containing one CDP-alcohol phosphatidyltransferase (CAP) domain--CLS_cap. In addition, only in eukaryotes the nascent CL is further remodeled to become mature CL, which generally contains the same fatty acids at sn-1, 2 sites in a molecule of a certain organism [14-16]. The indispensable eukaryotic CL maturation process and enzymes are as follows: nascent CL is deacylated to form monolysocardiolipin (MLCL), which is catalyzed by either of the two kinds of enzymes--CL-specific phospholipase (CLD1, YGR110W) identified in yeast [17] and calcium-independent phospholipase A_2 _(iPLA_2_) beta or gamma reported in *Drosophila *and rat [18,19]; MLCL is then reacylated by CoA-independent tafazzin (TAZ) [20] or acylCoA:lysocardiolipin acyltransferase 1 (ALCAT1) [21] to become mature CL. Through this process, a high degree of acyl chain symmetry in CL is established. In bacteria, there is not such a maturation process at all.

As seen above, the CL biosynthesis and maturation pathways in eukaryotes are distinct from those in bacteria. However, the simultaneous appearance of CL in both bacteria and eukaryotic mitochondria has been considered to be a line of evidence for the endosymbiotic origin of mitochondrion from bacteria [22,23]. According to the endosymbiosis theory, many mitochondrial properties such as energy metabolism including respiratory chain are inherited from the bacterial endosymbiont. But the above differences between mitochondria and bacteria make it uncertain whether this is true to CL biosynthesis pathway. Therefore, in fact how the eukaryotic CL biosynthesis and maturation pathways arise during the origin of eukaryotes from prokaryotes is still a mystery.

Moreover, CL was reported to be absent in some anaerobic protists such as *Giardia lamblia *[24] and *Trichomonas vaginalis *[25]. These organisms possess no canonical mitochondria but mitosomes or hydrogenosomes, which do not have electron transport chain (ETC), membrane potential, and proton-driven ATP generation [26]. The lack of mitochondria in *G*. *lamblia *was once taken as the main evidence by many authors to support this organism is the most primitive eukaryote diverging from the eukaryotic trunk before the emergence of mitochondria [27-29]. Therefore, whether the lack of CL in these 'amitochondriate' protists is due to their primitiveness or secondary degeneration is a question even relating to the early evolution of eukaryotes.

To study the origin and evolution of CL biosynthesis and maturation pathways, herein, phylogenetic distribution and phylogeny of the CL biosynthesis and maturation enzymes were investigated in diverse eukaryotes of the five supergroups: Opisthokonta, Amoebozoa, Archaeplastida, Chromalveolata, and Excavata, and diverse bacteria, and some interesting observations were obtained.

## Results

### Phylogenetic distribution of CL biosynthesis enzymes in eukaryotes and their similar sequences in bacteria

#### CL synthase (CLS)

Homologs of CLS_cap were identified in Opisthokonta (except the amitochondriate Microsporidia), Archaeplastida, and Stramenopiles (except *B*. *hominis*, which does not have genome database) of Chromalveolata (Table 1). The two supergroups and one subgroup contain all the multicellular eukaryotes (Animalia, Fungi, Planta, Chlorophyta, Rhodophyta, and Phaeophyceae) and some unicellular eukaryotes (protists). This means all the multicellular eukaryotes and only those unicellular eukaryotes that belong to the same supergroups (Opisthokonta and Archaeplastida) or subgroup (Stramenopiles of Chromalveolata) with these multicellular eukaryotes possess CLS_cap. Generally, each species has only one homolog, but a few of them such as *H*. *sapiens, M*. *musculus, C*. *elegans, D*. *melanogaster, S*. *purpuratus*, and *H*. *magnipapillata *have more than one copy (Additional file 1: Table S1). Multiple sequence alignments revealed most of these identified homologs possess the conserved amino acid residues and membrane-binding regions of the CLS_cap [30] (Additional file 2: Figure S1). Many (> 3,000) bacterial similar sequences were found in diverse bacteria following eukaryotic homologs in the hit list when searching against RefSeq_protein database when the cutoff E-value is 0.001, though most of them are annotated as CDP-diacylglycerol-glycerol-3-phosphate 3-phosphatidyltransferase (PGPS). To reduce computation burden, only those top hits (1,500 sequences, E < 1e-18) were included in the below analyses. Among them there are two previously reported CLS_cap from two actinobacteria [31], and according to our phylogenetic analysis, much more sequences from actinobacteria (88 of the 148 sequenced actinobacterial species) and some other bacteria including diverse proteobacteria and others are CLS_cap (data not shown). When these bacterial homologs were aligned to build HMM profile, and then the profile was used as query to search against all kinds of prokaryotic genomes, we also found only a small part of the surveyed bacteria (172 of the 1,375 bacteria), which are mainly proteobacteria, actinobacteria, and a few other bacteria, possess CLS_cap (data not shown).

**Table 1:** The phylogenetic distribution of CL biosynthesis and maturation enzymes of five eukaryotic supergroups

				CL biosynthesis			CL maturation			
				
		Organism		CLD			Step one		Step two	
							
				CLS_cap	CLS_pld		**iPLA2–γ**	iPLA2–β	ALCAT	TAZ
				
		*Homo sapiens*		**+**	**-**	**+**	**+**	**+**	**+**	**+**
		
		*Mus musculus*		**+**	**-**	**+**	**+**	**+**	**+**	**+**
		
	**Animalia**	*Xenopus laevis*		**+**	**-**	**+**	**+**	**+**	**+**	**+**
		
		*Gallus gallus*		**+**	**-**	**+**	**+**	**+**	**+**	**+**
		
		*Danio rerio*		**+**	**-**	**+**	**+**	**+**	**+**	**+**
		
		*Drosophila melanogaster*		**+**	**-**	**+**	**-**	**+**	**-**	**+**
		
**Opisthokonta**		*Caenorhabditis elegans*		**+**	**-**	**+**	**+**	**+**	**+**	**+**
		
		*Hydra magnipapillata*		**+**	**-**	**+**		**+**	**+**	**+**^a^
		
		*Strongylocentrotus purpuratus*		**+**	**-**	**+**	**+**	**+**	**+**	**+**
		
		*Schistosoma mansoni*		**+**	**-**	**+**		**+**	**+**	**+**
		
		*Ciona intestinalis*		**+**	**-**	**+**	**+**	**+**	**+**	**+**
	
	**Choanoflagellete**	*Monosiga brevicollis*		**+**	**-**	**+**	**-**	**-**	**+**	**+**
		
	**Fungi**		*Saccharomyces cerevisiae*	**+**	**-**	**+**	**-**	**-**	**+**	**+**
			
		**Ascomycota**	*Schizosaccharomyces pombe*	**+**	**-**	**+**		**-**	**+**	**-**
			
			*Aspergillus fumigatus*	**+**	**-**	**+**	**-**	**+**	**+**	**+**
		
		**Basidiomycota**	*Ustilago maydis*	**+**	**-**	**+**	**-**	**-**	**+**	**+**
			
			*Cryptococcus* *neoformans*	**+**	**-**	**+**	**-**	**-**	**+**	**+**
		
			*Antonospora locustae*	**-**	**-**	**-**	**-**	**-**	**-**	**-**
			
			*Encephalitozoon cuniculi*	**-**	**-**	**-**	**-**	**-**	**-**	**-**
			
		**Microsporidia**	*Enterocytozoon bieneusi*	**-**	**-**	**-**	**-**	**-**	**-**	**-**
			
			*Encephalitozoon intestinalis*	**-**	**-**	**-**	**-**	**-**	**-**	**-**

**Amoebozoa**	**Mycetozoa**	*Dictyostelium discoideum*		**-**	**+**	**-**	**-**	**+**	**+**	**+**
		
		*Dictyostelium purpureum*		**-**	**+**	**-**	**-**	**+**	**+**	**+**
	
	**Entamoebida**	*Entamoeba histolytica*		**-**	**-**	**-**	**-**	**-**	**-**	**-**
		
		*Entamoeba dispar*		**-**	**-**	**-**	**-**	**-**	**-**	**-**
		
		*Entamoeba invadens*		**-**	**-**	**-**	**-**	**-**	**-**	

**Archaeplastida**	**Planta**	*Arabidopsis thaliana*		**+**	**-**	**+**	**+**	**-**	**+**	**+**
		
		*Oryza sativa*		**+**	**-**	**+**	**+**	**-**	**+**	**+**
		
	**Chlorophyta**	*Chlamydomonas reinhardtii*		**+**	**-**	**+**	**+**	**-**	**-**	**+**
		
		*Ostreococcus lucimarinus*		**+**	**-**		**+**	**-**	**-**	**+**
		
		*Ostreococcus tauri*		**+**	**-**		**+**	**-**		**+**
		
		*Micromonas sp*.		**+**	**-**	**+**	**+**	**-**	**+**	**+**
	
	**Rhodophyta**	*Cyanidioschyzon merolae*		**+**	**-**	**+**	**+**	**-**	**-**	**+**
		
		*Galdieria sulphuraria *^#^		**+**^a^	**-**	**-**	**-**	**-**	**-**	**+**^a^

**Chromalveolata**	**Alveolata**	**Ciliata**	*Tetrahymena thermophila*	**-**	**+**	**+**	**-**	**-**	**-**	**-**
			
			*Paramecium tetraurelia*	**-**	**+**	**+**	**-**	**-**	**-**	**-**
		
		**Perkinsida**	*Perkinsus marinus*	**-**	**+**	**-**	**+**	**-**	**-**	**-**
		
			*Plasmodium knowlesi*	**-**	+	**-**	+	**-**	**-**	**-**
			
			*Plasmodium vivax*	**-**	**+**	**-**	**+**	**-**	**-**	**-**
			
			*Plasmodium faciparum*	**-**	**+**	**-**	**+**	**-**	**-**	**-**
			
		**Apicomplexa**	*Plasmodium chabaudi*	**-**	**+**	**-**	**+**	**-**	**-**	**-**
			
			*Plasmodium yoelli yoelii*	**-**	**+**	**-**	**+**	**-**	**-**	**-**
			
			*Cryptosporidium parvum*	**-**	**+**	**-**	**-**	**-**	**-**	**-**
			
			*Cryptosporidium hominis*	**-**	**+**	**-**	**-**	**-**	**-**	**-**
			
			*Cryptosporidium muris*	**-**	**+**	**-**	**-**	**-**	**-**	**-**
			
			*Toxoplasma gondii*	**-**	**+**	**-**	**+**	**-**	**-**	**-**
			
			*Babesia bovis *T2Bo	**-**	**+**	**-**	**+**	**-**	**-**	**-**
			
			*Theileria parva*	**-**	**+**	**-**	**+**	**-**	**-**	**-**
			
			*Theileria annulata*	**-**	**+**	**-**	**+**	**-**	**-**	**-**
	
			*Pythium ultimum*	**+**	**-**	**+**	**-**	**-**	**+**	**+**
			
			*Phytophthora sojae*	**+**	**-**	**+**	**-**	**-**	**+**	
			
		**Oomycetes**	*Phytophthora ramorum*	**+**	**-**	**+**	**-**	**-**	**+**	**+**
			
			*Phytophthora infestans*	**+**	**-**	**+**	**-**	**-**	**+**	**+**
			
	**Stramenopiles**		*Saprolegnia parasitica*	**+**	**-**	**+**	**-**	**-**	**+**	**+**
		
		Blastocystis hominis *		**-**	**-**	**-**	**-**	**-**	**+**	**+**
		
		**Bacillariophyta**	*Thalassiosira* *pseudonana*	**+**	**-**	**+**	**+**	**-**	**-**	**+**
		
			*Phaeodactylum tricornutum*	**+**^a^	**-**	**+**	**+**	**-**	**+**	**-**
		
		**Phaeophyceae**	*Ectocarpus siliculosus*	**+**	**-**	**+**	**+**	**-**	**+**	**+**

**Excavata**	**Kinetoplastids**	*Leishmania braziliensis*		**-**	**+**	**+**	**-**	**-**	**+**	**-**
		
		*Leishmania infantum*		**-**	**+**	**+**	**-**	**-**	**+**	**-**
		
		*Leishmania major*		**-**	**+**	**+**	**-**	**-**	**+**	**-**
		
		*Trypanosoma brucei*		**-**	**+**	**+**	**-**	**-**	**+**	**-**
		
		*Trypanosoma cruzi*		**-**	**+**	**-**	**-**	**-**	**+**	**-**
	
	**Heterolobosea**	*Naegleria gruberi*			+	**-**	+	**-**		+
	
	**Parabasalia**	*Trichomonas vaginalis*		**-**	**-**	**-**	**-**	**-**	**-**	**-**
	
	**Diplomonadida**	*Giardia lamblia*		**-**	**-**	**-**	**-**	**-**	**-**	**-**

+ = presence; - = absence; ^a^, homologs obtained by tblastn search rather than by blastp search as others; *, nr database rather than genome database was searched against for homologs; ^#^, EST database rather than genome database was searched against for homologs

Whereas, interestingly, in all the other two investigated eukaryotic supergroups and one subgroup, which all exclusively consist of unicellular eukaryotes (protists), including Amoebozoa (except the amitochondriate Entamoebida), Excavata (except the amitochondriate Parabasalia and Diplomonadida), and Alveolata in Chromalveolata, no CLS_cap but CLS_pld homologs were identified (Table 1). These homologs all contain the two conserved motifs which were proposed to be involved in phosphatidyl group transfer [32] (Additional file 3: Figure S2). Many (> 5,000, when E-value < 0.001) sequences annotated as CLS from diverse bacteria were also found to be top hits of CLS_pld. To investigate the distribution of CLS_pld in prokaryotes, a HMM profile built from seven genes whose CLS function were confirmed experimentally [33] was used as query to search bacterial genomes, CLS_pld homologs was found in most investigated bacteria (927 of the 1,375 bacteria). None type CLS is found in archaea.

None of the eukaryotes investigated contains the both types of CLS. Whereas, in all the amitochondriate protists mentioned above in brackets (e.g. Microsporidia, Entamoebida, Parabasalia, Diplomonadida), neither of the two types of CLS were found. No CLS were found in *B*. *hominis *yet, but this is probably due to its incomplete genome database.

#### CL-specific phospholipase (CLD)

Homologs were found in most genomes of four of the five eukaryotic supergroups except Amoebozoa, but within the four supergroups some subgroups or species such as Microsporidia, *Ostreococcus, G*. *sulphuraria*, Perkinsida, Apicomplexa, *B*. *hominis*, Heterolobosea, Parabasalia, and Diplomonadida do not have the homolog yet (Table 1). Two typical motifs ("GXSXG" and "HX4D") of CLD [17], which are considered to function as lipase and acyltransferase, respectively, were found in almost all of these identified homologs (Additional file 4: Figure S3). Many (> 5,000, when E-value < 0.001) bacterial similar sequences were also found following the eukaryotic homologs in the hit list, but most of them were annotated as "alpha/beta hydrolase" or "hypothetical protein". We only choose those very close to eukaryotic sequences in the hit list for the below phylogenetic analyses.

#### Calcium-independent phospholipase A2 (iPLA2)

As the hits of iPLA_2 _beta and gamma mixed together in the hit list due to high sequence similarity between the two enzymes, they were discriminated according to the below phylogenetic analyses. It was found homologs of iPLA_2 _gamma exist in most genomes of four of the five supergroups (except Amoebozoa) and homologs of iPLA_2 _beta were found in all animals and a fungus in Opisthokonta and two species in Amoebozoa. None homologs of the two iPLA_2 _were found in many subgroups and species, such as Choanoflagellate, most fungi (except *A*. *fumigatus*), Entamoebida, *G*. *sulphuraria*, Ciliata, Cryptosporidium, Oomycetes, *B*. *hominis*, Parabasalia, and Diplomonadida. But many other fungi not listed in Table 1 were found to have iPLA_2 _homologs when searching against RefSeq_protein database. Some organisms possess multiple homologs (Additional file 1: Table S1). Most of the identified homologs possess the two conserved segments which are the features of iPLA_2 _[34] (Additional file 5: Figure S4). Many bacterial similar sequences annotated to be "patatin" were found following these eukaryotic homologs in the hit list, and only those top hits (> 500 sequences when E-value < 0.001 for each query) were picked and supplied to the below phylogenetic analyses.

#### acyl-CoA:lysocardiolipin acyltransferase 1 (ALCAT)

Besides annotated ALCAT, other eukaryotic enzyme homologs such as "1-acylglycerol-3-phosphate O-acyltransferase (AGPAT) 3, 4, 5" and "lysophosphatidylglycerol acyltransferase (LPGAT)" were also found in the genomes of all five supergroups when searching against the RefSeq_protein database. Because of the high sequence similarities among them, their identities were further determined by the below phylogenetic analyses. No homolog was found in several subgroups and species including *D*. *melanogaster*, Microsporidia, Entamoebida, most Chlorophyta (except *M*. *sp*.), Rhodophyta, Alveolata, *T*. *pseudonana*, Heterolobosea, Diplomonadida, and Parabasalia (Table 1). Many (> 1,000, when E-value < 0.001) bacterial similar sequences were also found following the above eukaryotic homologs in the hit list. Their relationship with eukaryotic ALCAT homologs was determined by the below phylogenetic analyses.

#### Tafazzin (TAZ)

Homologs were found in all the five supergroups, but not found in several subgroups and species such as Microsporidia, Entamoebida, Alveolata, Kinetoplastids, Parabasalia, Diplomonadida, *S*. *pombe, P*. *sojae*, and *P*. *tricornutum *(Table 1). Bacterial sequences were also found after eukaryotic TAZ homologs in the hit list, and were mostly annotated as "acyltransferase". But they have very low sequence similarities with eukaryotic TAZ homologs, and our preliminary phylogenetic analysis does not support they have close relationship with eukaryotic TAZ, thus they were not included in the further analyses.

Briefly, the distribution of the maturation pathway enzymes can be summarized as the following three conditions: 1) not any enzymes exist in Microsporida, Entamoebida, Cryptosporidium, Parabasalia, and Diplomonadida; 2) there are only one or two enzymes in some protists, including *G*. *sulphuraria*, Alveolata (except Cryptosporidium), and *B*. *hominis*, they are unable to form the complete two-step maturation pathway in these protists; 3) all the other eukaryotes possess most of the enzymes, which can form the complete two-step maturation pathway.

### Phylogeny of CL biosynthesis enzymes

As the Maximum Likelihood (ML) and Bayesian trees showed similar topologies, here we chose the Bayesian tree as a representative with the bootstrap values of ML tree also on the tree (As for the following other enzymes, the similar results were obtained, and so Bayesian trees were also chosen as representatives).

**Figure 2:**
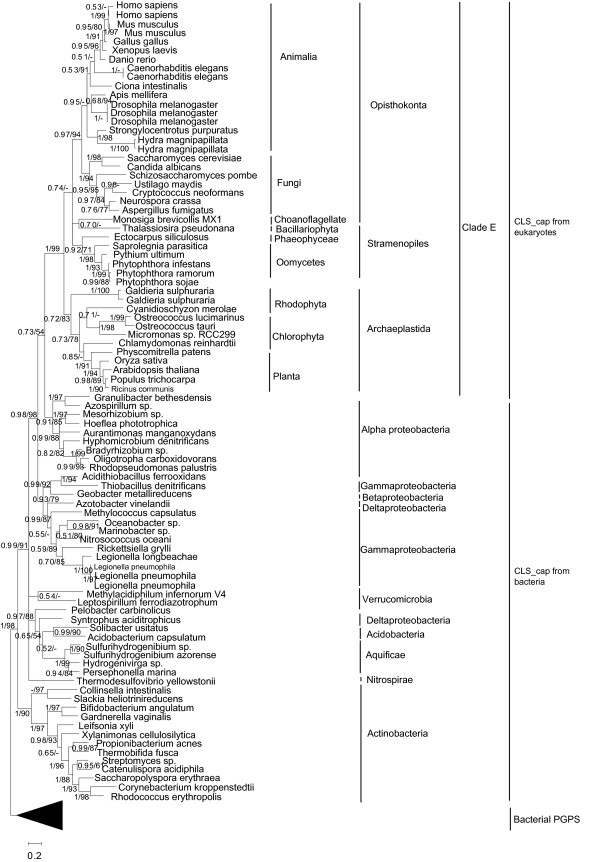
**Phylogeny of all CLS_cap from eukaryotes and bacteria, and PGPS homologs of bacteria**. Tree was inferred using MrBayes 3.12 on aligned amino acid dataset. Numbers at the nodes correspond to Bayesian posterior probabilites ≥ 0.50 (at the left of slashes) and the bootstrap value of ML tree (at the right of slashes). Scale bar indicates number of change per site. Bacterial PGPS are condensed as a triangle clade and rooted as outgroup.

On the CLS_cap phylogenetic tree (Figure 2, for the ML tree please see Additional file 6: Figure S5), all the identified homologs from eukaryotes are recovered into a highly supported big monophyletic clade (Clade E). Within this clade, homologs from Opisthokonta, Archaeplastida, and Stramenopiles of Chromalveolata form three subclades with high support values, and within these subclades many groups corresponding to their source lineages were also recovered. Furthermore, multiple homologs from a species always cluster together firstly, suggesting they are the products of species-specific gene duplication. A clade consisting of all homologs from alpha proteobacteria was recovered to be the closest sistergroup of the Clade E with a moderate support value (0.73/54) with all the homologs of other diverse bacteria being its outgroups. Among these outgroups, the actinobacterial clade, which contains the two previously reported CLS_cap identified from two actinobacteria [31], is the outmost group, suggesting all the homologs of these outgroups are CLS_cap. Finally, PGPS from diverse bacteria form an outgroup of all the above clades. Therefore, our results suggest besides in actinobacteria as reported previously, CLS_cap might have already emerged in some other bacteria including diverse proteobacteria and others, and eukaryotes might acquire their CLS_cap from alpha proteobacteria.

**Figure 3:**
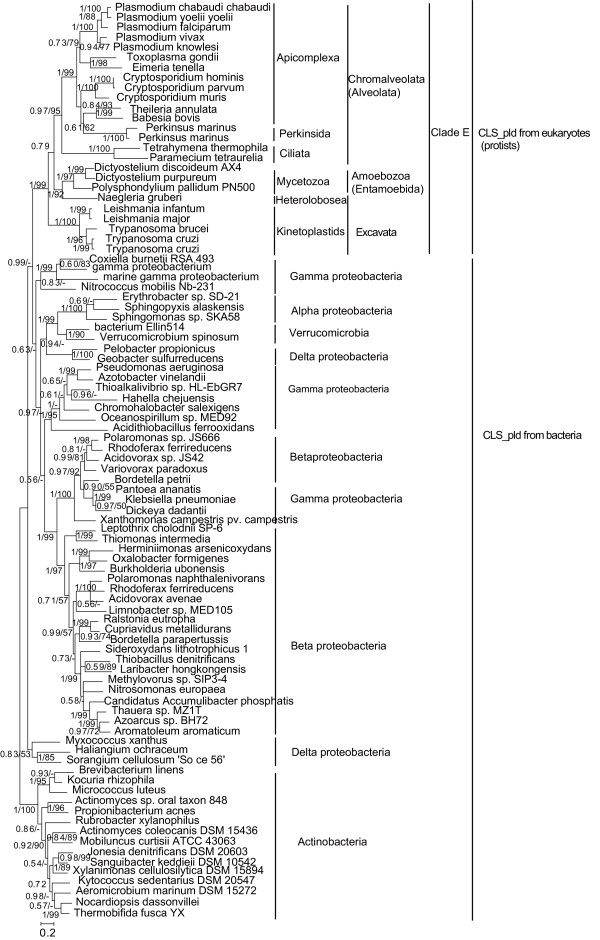
**Phylogeny of all CLS_pld from the eukaryotes (which all are protists) and diverse bacteria**. The tree was inferred using MrBayes 3.12 on 356 aligned amino acids. The tree is illustrated using the same conventions as in Figure 2.

On the CLS_pld phylogenetic tree (Figure 3), all the identified homologs from eukaryotes are also recovered into a highly supported big monophyletic clade (Clade E). Within this clade, homologs form three subclades almost corresponding to their three source supergroups--Alveolata of Chromalveolata, Amoebozoa, and Excavata, and within these subclades homologs also form groups corresponding to their source lineages (e.g. Apicomplexa, Perkinsida, and Ciliata). However, Clade E does not show any particular close correlations with those similar sequences from any current bacterial lineages. These results suggest that all the CLS_pld from the eukaryotes (which are exclusively unicellular organisms, protists) of the three eukaryotic supergroups have a common ancestor, which does not fall into any of the present bacterial lineages.

### Phylogeny of CL maturation enzymes

Due to their very low sequence similarities with TAZ and ALCAT, bacterial similar sequences of these two enzymes were not included in the final phylogenetic analysis. The obtained four phylogenetic trees (Additional file 7: Figure S6, Additional file 8: Figure S7, Additional file 9: Figure S8, Additional file 10: Figure S9 and Additional file 11: Table S2) showed: 1) all the eukaryotic homologs of each enzyme cluster together firstly with high support values, none of these enzymes show a close relationship with any particular bacterial similar sequences, and the low support values also do not support they have direct phylogenetic correlations with any bacterial sequences, suggesting they are not inherited from bacterial ancestors directly but arose after the emergence of eukaryotes, and each of these enzymes in all eukaryotes has a common ancestor which have might already emerged in the last eukaryotic common ancestor (LECA) of the five supergroups; 2) homologs of each enzymes from a common supergroup or lineage (e.g. Animalia, Fungi, Oomycetes and Planta) do not form a common clade corresponding their source supergroup or lineage but usually form two or more separated clades, and alternative trees constraining them as monophyly were rejected significantly (Additional file 11: Table S2), suggesting gene duplication and loss occurred frequently on these enzymes at different lineage levels. Moreover, as for ALCAT, firstly, all the homologs form a sistergroup to AGPAT 3/4 clade, suggesting ALCAT arose through gene duplication and divergence with the enzyme AGPAT 3/4. This means gene duplication and divergence also have ever occurred between ALCAT and AGPAT 3/4 during the origin of ALCAT. What is more, multiple copies of homologs of each of these enzymes from a species generally clustered together, suggesting gene duplication of these enzymes continues occurring relatively recently in some species.

## Discussion

### The origin and evolution of CL biosynthesis pathways in eukaryotes

As mentioned above, CL is biosynthesized by two distinct synthases--CLS_cap and CLS_pld. The two types of enzymes belong to two distinct protein families without any primary sequence similarity between them [16]. Generally, it is considered eukaryotes have CLS_cap and bacteria CLS_pld. However, our investigation revealed although most bacteria possess CLS_pld, some kinds of bacteria including actinobacteria, proteobacteria, and some others, bear CLS_cap, suggesting CLS_cap has already arisen in some bacteria actually; in eukaryotes, all the multicellular organisms and only those unicellular organisms (protists) which belong to the same supergroups or subgroup with these multicellular organisms possess CLS_cap. Our phylogenetic analysis further showed all the CLS_cap in these eukaryotes have the closest relationship with those of alpha proteobacteria. Since alpha proteobacteria is generally considered to be the endosymbiotic ancestor of mitochondrion [35-37], then the CLS_cap pathway in these eukaryotes most probably originated from alpha proteobacteria through the mitochondrial endosymbiotic event. This is inconsistent with the previous postulation that eukaryotic CLS originated from the prokaryotic type PGPS which existed in ancestral eukaryotes [38].

On the other hand, our investigation revealed all the other eukaryotes whose supergroups or subgroup consist exclusively of unicellular eukaryotes (protists) possess CLS_pld. Among these eukaryotes a few lineages such as Trypanosoma, Leishmania, Theileria, Plasmodium, Cryptosporidium and Dictyostelium had previously been reported to have CLS_pld by other authors, and this condition was explained as an evolutionary survival of the prokaryotic reaction for CL formation into the eukaryotic kingdom [38]. Actually, CL was reported to really exist in these eukaryotes such as *D*. *discoideum, T*. *thermophila, P*. *tetraurelia, P*. *marinus *and *T*. *cruzi *[39-43]. But, according to our present work, since 1) CLS_pld is widely distributed in so many kinds of protists (only with the exception of those protists in Supergroup Opisthokonta, Archaeplastida, and Stramenopiles of Supergroup Chromalveolata), and forms a complementary distribution with the CLS_cap within the entire eukaryote Domain (mainly within protists); 2) on the phylegenetic tree, all the CLS_pld from different eukaryotes (protists) were clustered together as a common clade, without showing close relationship with the CLS_pld from any particular extant bacterial lineages, suggesting they have a common ancestor which is probably very ancient and is not kept in any extant bacterial lineages without obvious changes, then these CLS_pld in eukaryotes can not be a secondary acquisition by independent horizontal gene transfer (HGT) from different bacteria in different protist lineages, but must have be inherited from a common ancestor of these eukaryotes. Because 1) such a common ancestor can only be the last eukaryotic common ancestor (LECA) or the first eukaryotic common ancestor (FECA); 2) most bacteria (except most proteobacteria and actinobacteria, which bear CLS_cap pathway) possess CLS_pld pathway, and the emergence of CLS_cap in partial bacteria might occur much later than CLS_pld; 3) the common ancestor of these eukaryotic CLS_pld can not be found in extant bacteria as that of eukaryotic CLS_cap, so the acquisition of these eukaryotic CLS_pld might occurred very anciently (probably earlier than the endosymbiotic origin of mitochondria from alpha proteobacteria). Therefore, it is most probably that the FECA inherited the CLS_pld pathway from a ancient bacterium such as the bacterial partner according to the "fusion hypothesis" [44], or the proto-eukaryote derived from bacteria according to the 'phagotrophy hypothesis' [45], or the bacteirium related to the origin of the nucleus according to the 'endosymbiosis hypothesis'[46-48].

Neither CLS_cap nor CLS_pld was found in all the investigated amitochondriate protists, inspite of which eukaryotic supergroup (Opisthokonta, Amoebozoa, or Excavata) these protists belong to. This is consistent with the lack of CL in these organisms such as *G*. *lamblia, T*. *vaginalis*, and *E*. *cuniculi *[24,25,49]. Since both bacteria and all the other eukaryotes have CL and the corresponding CL biosynthesis pathways, the absence of either of the two CL biosynthesis pathways in these amitochondriate protists must be the results of secondary loss due to their degeneration of mitochondria. Consistently, it was showed anaerobic prokaryotes lack CL, and anaerobic condition can cause the decrease of CL in contrast to aerobic in yeast [50,51]. The existence of CL in a relative of *T*. *vaginalis*-- *Tritrichomonas foetus *[23] further support such a secondary loss once occurred at least in *T*. *vaginalis*. The lack of either type of CLS in *B*. *hominis *might also due to its lack of mitochondria or incomplete genome database.

**Figure 4:**
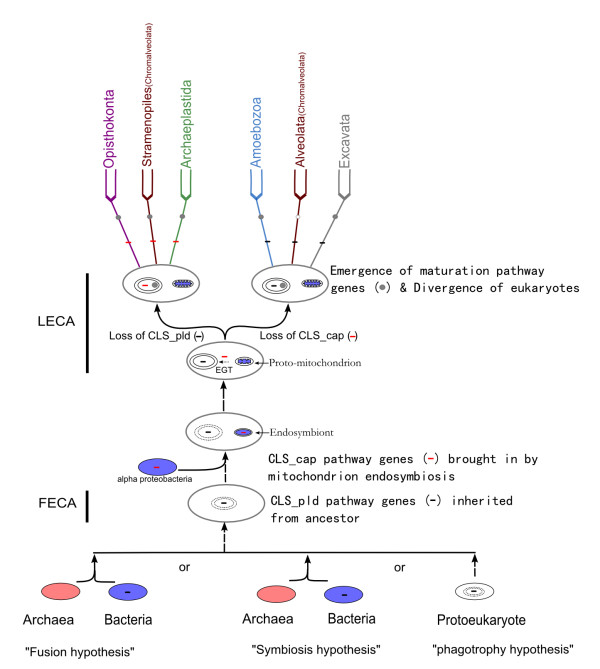
**An evolutionary route chart of CL biosynthesis and maturation pathways in eukaryotes**. Short black bar, CLS_cap gene; Short red bar, CLS_pld gene; Grey dot, CL maturation pathway genes; Hollow dot, absence of CL maturation pathway. EGT, endosymbiotic gene transfer from proto-mitochondrion to nucleus.

Considering the distinctive difference of phospholipid between archaea and bacteria and eukaryotes [52], and the absence of either type of CLS in archaea, it is reasonable to postulate archaea may not contribute to the origin of eukaryotic CL biosynthesis. Therefore, based on the above analyses, we can propose a evolutionary scenario about the CL biosynthesis pathway in eukaryotes as follow (Figure 4): in the process of the origin and evolution of eukaryotes, the FECA inherited the CLS_pld pathway from its bacterial ancestor, which is probably the bacterial partner according to any of the hypotheses about eukaryote evolution such as the 'fusion hypothesis', the 'phagotrophy' hypothesis and the 'endosymbiosis hypothesis'; later, when the FECA evolved into LECA, the endosymbiotic origin of mitochondrion brought in another CL synthase--CLS_cap, which had arisen in the endosymbiotic bacteria--alpha proteobacteria; then, in those LECA individuals which would evolve into those unicellular eukaryote lineages (e. g. Chonanoflagellates, Chlorophyta) from which multicellular eukaryotes (e. g. Animalia and Fungi in Opisthokonta, Archaeplastida, and Phaeophyceae in Chromalveolata) could arise, the endosymbiotic-original CLS_cap gene was transferred into the nuclear genome of the host cell, and the previous CLS_pld pathway was substituted, while in the other LECA individuals which would just evolve into the other unicellular protist lineages (e. g. Amoebozoa, Alveolata of Chromalveolata, and Excavata) from which no multicellular eukaryotes would arise, the previous CLS_pld was retained and the endosymbiotic-original CLS_cap was lost; in the amitochondriate protists (including Microsporidia) the CL biosynthesis pathway (either CLS_pld or CLS_cap) was secondly totally lost due to their secondary degeneration of mitochondria.

### The origin and evolution of CL maturation pathway in eukaryotes

The eukaryotic CL maturation pathway consists of two steps, and altogether five enzymes have been previously identified to participate in this process in different eukaryotes.

CL maturation is indispensable in higher eukaryotes though the purpose of this process is not very clear. Our phylogenetic analyses indicated all maturation enzymes arise after the emergence of eukaryotes, and might have already emerged prior to the divergence of all the eukaryote supergroups. Except ALCAT seems to arise through gene duplication and divergence of other existing enzyme (AGPAT 3/4), the origins of the other enzymes are not clear yet.

Our phylogenetic analyses also indicated gene duplication and gene loss occurring frequently at different lineage levels in the evolution of maturation pathways. These gene duplications and losses result in a patchy distribution of the maturation pathway enzymes in diverse eukaryotes, increasing the diversity of the pathway. Different enzymes or multiple homologs in the same step of the pathway can widen the recognition of substrates carrying different fatty acid substituents, and iPLA_2 _beta and gamma are just such a reported example for this [53]. Such a condition for the maturation pathway might be the results of adaptive evolution for coping with the complicated cellular process in various eukaryotic cells.

In the present work, we found except some unicellular eukaryotes including all the amitochondriate protists (Microsporidia, Entamoebida, Parabasalia and Diplomonadida), all Alveolata in Chromalveolata, and a few other species (e. g. *G*. *sulphuraria, B*. *hominis*), all the other eukaryotes, which distribute in all the five eukaryotic supergroups, either unicellular or multicellular, either parasitic or free-living, possess a complete CL maturation pathway by having at least one enzyme for each step of the pathway. The absence of the complete pathway in *B*. *hominis *and *G*. *sulphuraria *might be caused by their incomplete databases, and the lack in other protists are probably due to various secondary losses, because 1) each enzyme of pathway from various lineages form a monophyly on the phylogenetic trees, 2) their close relatives have this pathway, and 3) some, though not a complete set, of the enzymes of the pathway, appear in some of these protists. The totally absence of this pathway in amitochondriate protists (without any enzymes of this pathway) is consistent with the lack of typical mitochondria and CL in these protists, and must be due to the degeneration of mitochondria in them. Whereas, the presence of partial of the enzymes of this pathway in some protists (e.g. Ciliata, Perkinsida, most Apicomplexa) might suggest their maturation pathways are in the process of losing or the enzymes left might have other functions.

### Implications to the evolution of eukaryotes and the classification of the five eukaryotic supergroups

According to our above analysis about the phylogenetic distribution and the phylogeny of the two types of CLS in eukaryotes, the acquisition of CLS_cap pathway through mitochondrial endosymbiosis might have offered some potential for the evolution of multicellularity. Because the CLS_pld pathway exists exclusively in unicellular eukaryotes (protists), while the CLS_cap pathway is distributed in all the multicellular organisms and only those unicellular eukaryotes (protists) that belong to the same supergroups or subgroup with these multicellular organisms. Therefore, for the first time, our work implies the endosymbiotic event of alpha proteobacteria not only led to the origin of mitochondria, but also might affect the subsequent evolution of eukaryotes such as the evolution of multicellularity, which may depend on what kinds of genes of the endosymbiont are transferred into the host nucleus and thus what kinds of endosymbiotic relationships are established.

The classification and relationships of the five eukaryotic supergroups are still under controversial now [54-57]. In the present work, it was showed the CL biosynthesis and maturation pathways are very different between the two subgroups in Supergroup Chromalveolata--Stramenopiles possess the CLS_cap pathway and a complete maturation pathway, while Alveolata bear the CLS_pld pathway and not a complete maturation pathway (due to completely lacking the second step). Therefore, the classification putting these two subgroups into a common supergroup may be unreasonable.

Amitochondriate protists were once thought as the most primitive extant eukaryotes because of their lack of mitochondrion and other primitive characteristics [28,29,58]. However, recently, accumulating molecular evidence and the identification of atypical mitochondria-- mitosome or hydrogenosome--in these organisms argued they might once possess mitochondria [59-62]. Our investigation indicates the absence of CL biosynthesis and maturation pathways in these amitochondriate protists might be due to secondary losses. Thus, these atypical mitochondria in these amitochondriate protists might also result from degeneration of their once-existent typical mitochondria.

## Conclusions

We propose that the FECA inherited the CLS_pld pathway from its bacterial ancestor (which could be the bacterial partner according to the 'fusion hypothesis' or the 'phagotrophy hypothesis' or the 'endosymbiosis hypothesis' about the origin of eukaryotes from prokaryotes); later, when the FECA evolved into the last eukaryote common ancestor (LECA), the endosymbiotic mitochondria (alpha proteobacteria) brought in another pathway--CLS_cap pathway, and then in some LECA individuals the CLS_cap pathway substituted the previous CLS_pld pathway, and these LECA would evolve into the protist lineages from which multicellular eukaryotes could arise, while in the other LECA individuals the previous CLS_pld pathway was kept and the CLS_cap pathway was lost, and these LECA would evolve into the current protist lineages that possess the CLS_cap pathway. Besides, our work indicated CL maturation pathway arose after the emergence of eukaryotes probably through mechanisms such as the duplication of other already-existent genes, and gene duplication and loss occurred frequently at different lineage levels, increasing the diversity of the pathway probably so as to fit the complicated cellular process in various cells. On the other hand, our work implies what kind of the endosymbiotic relationship is established during the evolutionary origin of mitochondrion in early eukaryotes might affect the subsequent evolution of multicellularity; the classification putting Stramenopiles and Alveolata together to form Chromalveolata may be unreasonable; the absence of CL synthesis and maturation pathways in amitochondriate protists is most probably due to secondary degeneration.

## Methods

### Organisms

The following organisms with genome or expressed sequence tag (EST) databases were taken as representatives of the five eukaryotic supergroups in this study: 1) Opisthokonta: Animalia (vertebrates: *Homo sapien, Mus musculus, Xenopus laevis, Gallus gallus, Danio rerio*; invertebrates: *Drosophila melanogaster, Caenorhabditis elegans, Hydra magnipapillata, Strongylocentrotus purpuratus, Schistosoma mansoni, Ciona intestinalis*), Choanoflagellate (*Monosiga brevicollis*), and Fungi (Ascomycota [*Saccharomyces cerevisiae, Schizosaccharomyces pombe, Aspergillus fumigatus*], Basidiomycota [*Ustilago maydis, Cryptococcus neoformans*], Microsporidia [*Encephalitozoon cuniculi, E. intestinalis, Enterocytozoon bieneusi*]), 2) Amoebozoa (Mycetozoa [*Dictyostelium discoideum, D. purpureum*], Entamoebida [*Entamoeba histolytica, E. dispar, E. invadens*]), 3) Archaeplastida (Planta [*Arabidopsis thaliana, Oryza sativa*], Chlorophyta [*Chlamydomonas reinhardtii, Ostreococcus lucimarinus, O. tauri, Micromonas sp*. RCC299], Rhodophyta [*Cyanidioschyzon merolae, Galdieria sulphuraria*]); 4) Chromalveolata (Alveolata (Ciliata [*Tetrahymena thermophila, Paramecium tetraurelia*], Perkinsida [*Perkinsus marinus*], Apicomplexa [*Plasmodium knowlesi, P. vivax, P. faciparum, P. chabaudi, P. yoelli yoelii, Cryptosporidium parvum, C. hominis, C. muris, Toxoplasma gondii, Babesia bovis, Theileria parva, T. annulata*]), Stramenopiles (*Blastocystis hominis*, Oomycetes [*Pythium ultimum *BR144, *P. sojae, P. ramorum, P. infestans, Saprolegnia parasitica *CBS 22], Bacillariophyta [*Thalassiosira pseudonana *CCMP1335, *Phaeodactylum tricornutum *CCAP1055/1], Phaeophyceae [*Ectocarpus siliculosus*])), 5) Excavata (Heterolobosea [*Naegleria gruberi*], Kinteoplastids [*Leishmania braziliensis, L. infantum, L. major, Trypanosoma bruzi, T. cruzi*], Diplomonadida [*Giardia lamblia *str. WB], Parabasalia [*Trichomonas vaginalis*]) (Additional file 12: Table S3). Their genome or EST databases were downloaded. In addition, other eukaryotes and various prokaryotes were also included in this study when BLASTp searching against the Refseq_protein database (Release 44, January, 2011) of NCBI database.

### CL biosynthesis and maturation pathway gene collection and identification

All the reviewed eukaryotic CLS sequences (Q07560, O01916, Q8MZC4, Q9UJA2, Q80ZM8, Q5U2V5, and B6TPV7) and bacterial CLS sequences (127 sequences, their accession ID and sequences can be obtained from the authors upon request), and reviewed TAZ sequences (Q9V6G5, Q16635, Q6IV77, Q06510, Q6IV84, Q6IV76, Q6IV83, Q6IV82, Q6IV78, and Q54DX7) were downloaded from Uniprot. As only a few reviewed CLD1, PLA2 and ALCAT are available in Uniprot, the curated orthologs of CLD1 (K13535) and ALCAT (K13513) were downloaded from KEGG database; As for iPLA_2 _beta (CG6718) and gamma (Q9NP80), their putative orthologs (beta: 15 sequences; gamma: 14 sequences. Their accession ID and sequences can be obtained from the authors upon request.) were retrieved from KEGG SSDB database (hits with best-best relationship and identity > 0.5). These obtained sequences were aligned by MUSCLE, v 3.8.31 [63]. Then, HMM profile of each enzyme was build and calibrated from their multiple aligned sequences by HMMER package (v3.0) with default parameters. Finally, the obtained profiles were used as queries to search against genome databases of those organisms mentioned above and ResSeq_protein databases by using hmmsearch. The obtained similar sequences with high E-value were further analyzed by PFAM to confirm whether they are really homologs. To exclude repeat "ANK" domain of PLA_2 _beta (CG6718 and its orthologs), corresponding N-terminal region were removed according to the annotation of PFAM database before hmm profiles building. If no similar sequence was detected for a certain species, then its non-redundant (nr) protein and nucleotide database and genome database online were searched against by using BLASTP or tBLASTn program independently. The EST database of *G*. *sulphuraria *was searched against by using tBLASTn program.

Bacterial similar sequences of each of these enzymes were also collected during searching against RefSeq_protein database. As many bacterial similar sequences were found under the cutoff E-value 0.001, they were collected as many as possible at first and then only a subset of them, determined by using preliminary phylogeny analyses were kept for the further analyses.

### Phylogenetic analyses

In order to infer the origin of eukaryotic CL biosynthesis and maturation enzymes, all the sequences obtained above were used for the following phylogenetic analyses.

Multiple alignment of each dataset was initially carried out using MUSCLE, version 3.8.31 [63]. Nonhomologous insertions and sequence characters that could not be aligned with confidence were removed manually. Only unambiguously aligned sites were used for phylogenetic analyses.

Phylogenetic trees were inferred using maximum likelihood (ML) and Bayesian methods. ML trees were inferred with FastTree 2.1 [64] using default CAT model and other settings. MrBayes 3.1.2 [65] was used to perform parallel Bayesian analyses with four incrementally heated Markov chains, sampled every 1,000 generations with the temperature set to 0.5. Among-site substitution rate heterogeneity was corrected with an invariable and eight Γdistributed substitution rate categories and the WAG model for amino acid substitutions [66], abbreviated herein as WAG+I+8 G. Two separate runs were performed to confirm the convergence of the chains. The average standard deviation of split frequencies and the potential scale reduction factor convergence diagnostic were used to assess the convergence of the 2 runs. Trees below the observed stationarity level were discarded, resulting in a 'burnin' that comprised 25% of the posterior distribution of trees. The 50% majority-rule consensus tree was determined to calculate the posterior probabilities for each node.

Prior to the above phylogenetic analyses, usually the large data sets including much more bacterial similar sequences were applied for preliminary analysis by using FastTree 2.1 with default parameters, and then only the sub-datasets including eukaryotic sequence data and the closest relationship with eukaryotes on the preliminary trees were picked out and subjected to the further analysis.

### Tree topology tests

To assess the significance of gene duplication in each of the maturation pathway enzymes, alternative trees constraining two or more separate subclades of a certain lineage as a monphyly were obtained by 20 searches using RAxML [67] with the models mentioned above. The best-scoring ML tree from each constraint tree search was then compared with the Bayesian tree. Site likelihoods were calculated in RAxML (-f g option) under the GTRGAMMA model of sequence evolution. The Approximately Unbiased (AU) test was performed using CONSEL 0.1 k [68].

## Abbreviations

CL: Cardiolipin; CLS: CL synthase; LECA: Last eukaryotic common ancestor; CLD: CL-specific phospholipase; PG: Phosphatidylglycerol; CDP-DAG: Cytidine diphosphate diacylglycerol; PLD: Phospholipase D; CAP: CDP-alcohol phosphatidyltransferase; MLCL: Monolysocardiolipin; iPLA_2_: Calcium-independent phospholipase A_2_; TAZ: Tafazzin; ALCAT1: Acyl-CoA:lysocardiolipin acyltransferase 1; AGPAT: 1-acylglycerol-3-phosphate O-acyltransferase; ETC: Electron transport chain.

## Competing interests

The authors declare that they have no competing interests.

## Authors' contributions

HFT conceived the project, carried out phylogenetic distribution investigation and phylogenetic analyses, JMF carried out database searches and phylogenetic analyses, and JFW supervised the work. HFT and JFW wrote the manuscript. All authors read and approved the final manuscript.

## Supplementary Material

Additional file 1 Additional file 1. Identified homologs involved in CLS synthesis and maturation pathways in eukaryotes. Click here for file

Additional file 2 **Figure S1**. The alignment of CLS_cap of eukaryotes (part). Conserved six membrane-binding regions are designated as I-VI and conserved amino acid residues among CAP family are boxed. Amino acid positions are numbered relative to the *Monosiga brevicollis *ortholog. # below the alignment indicates the amino acid residues that are specific for CL synthases. Click here for file

Additional file 3 **Figure S2**. The identified conserved motifs (the boxed regions) of CLS_pld from mitochondriate protists. Amino acid positions are numbered relative to the *Plasmodium knowlesi *ortholog (gi: 221058144). Click here for file

Additional file 4 **Figure S3**. The identified conserved motifs of CLD of eukaryotes. Two conserved regions that might function as lipase and acyltransferase motifs are boxed. Amino acid positions are numbered relative to the *Phytophthora ramorum *ortholog (id: Pr_95977T0). Click here for file

Additional file 5 **Figure S4**. The identified conserved motifs of iPLA_2 _of eukaryotes. Two conserved segments among iPLA2 are indicated by lines marked on the head. Conserved Ser and Asp residues that form a catalytic dyad, and the Gly-Gly dipeptide of the oxyanion hole are indicated by asterisks. Click here for file

Additional file 6 **Figure S5**. The ML phylogenetic tree of all the CLS_cap from eukaryotes and bacteria, and PGPS homologs of bacteria, which is corresponding to the Bayesian tree of Figure 2. Click here for file

Additional file 7 **Figure S6**. Phylogeny of eukaryotic homologs of CLD and bacterial similar sequences. The tree was constructed by using MrBayes 3.1.2, and is illustrated using the same conventions as Figure 1. The monophyly constraint of Fungi (Fungi1+Fungi2) passed the AU test, suggesting they might be obtained through lineage-specific gene duplication. Click here for file

Additional file 8 **Figure S7**. Phylogeny of iPLA_2 _and related bacterial similar sequences. The tree was constructed by using MrBayes 3.1.2, and is illustrated using the same conventions as Figure 1. The rejection of monophyly hypothesis of Animalia (Animalia1+Animalia2) by AU test (0.048) argues that iPLA_2 _beta and gamma diverged in the ancestor of Animalia though it's hard to determine the time. Click here for file

Additional file 9 **Figure S8**. Phylogeny of ALCAT and AGPAT 3/4. The tree was constructed by using MrBayes 3.1.2, and is illustrated using the same conventions as Figure 1. AGPAT 3/4 were rooted as outgroup based on our preliminary analyses. The tree is illustrated using the same conventions as in Figure 1. Alternative trees constraining all Stramenopiles as monophyly were rejected, suggesting gene duplication occurred in the ancestor of Stramenopiles. Click here for file

Additional file 10 **Figure S9**. Phylogeny of the TAZ and bacterial similar sequences. The tree was constructed by using MrBayes 3.1.2, and is illustrated using the same conventions as Figure 1. Hypothetical trees constraining all Archaeplastids as monophyly were rejected, suggesting gene duplication occurred in the ancestor of Archaeplstids. Click here for file

Additional file 11 Additional file S11. Comparision between Bayesian tree and alternative topologies Click here for file

Additional file 12 Additional file S12. The download sites of eukaryotic genomes or EST database included in the analyses Click here for file

## References

[B1] Zhang M Mileykovskaya E Dowhan W Cardiolipin is essential for organization of complexes III and IV into a supercomplex in intact yeast mitochondria J Biol Chem 2005 280 33 29403 29408 10.1074/jbc.M504955200 15972817 PMC4113954

[B2] Joshi AS Zhou JM Gohil VM Chen SL Greenberg ML Cellular functions of cardiolipin in yeast Biochimica Et Biophysica Acta-Molecular Cell Research 2009 1793 1 212 218 10.1016/j.bbamcr.2008.07.024 PMC2788820 18725250

[B3] Koshkin V Greenberg ML Cardiolipin prevents rate-dependent uncoupling and provides osmotic stability in yeast mitochondria Biochem J 2002 364 317 322 11988106 10.1042/bj3640317 PMC1222575

[B4] Jiang F Ryan MT Schlame M Zhao M Gu ZM Klingenberg M Pfanner N Greenberg ML Absence of cardiolipin in the crd1 null mutant results in decreased mitochondrial membrane potential and reduced mitochondrial function J Biol Chem 2000 275 29 22387 22394 10.1074/jbc.M909868199 10777514

[B5] Yankovskaya V Horsefield R Tornroth S Luna-Chavez C Miyoshi H Leger C Byrne B Cecchini G Iwata S Architecture of succinate dehydrogenase and reactive oxygen species generation Science 2003 299 5607 700 704 10.1126/science.1079605 12560550

[B6] Jormakka M Byrne B Iwata S Formate dehydrogenase--a versatile enzyme in changing environments Curr Opin Struct Biol 2003 13 4 418 423 10.1016/S0959-440X(03)00098-8 12948771

[B7] Arias-Cartin R Grimaldi S Pommier J Lanciano P Schaefer C Arnoux P Giordano G Guigliarelli B Magalon A Cardiolipin-based respiratory complex activation in bacteria Proc Natl Acad Sci USA 2011 108 19 7781 7786 10.1073/pnas.1010427108 21518899 PMC3093509

[B8] McAuley KE Fyfe PK Ridge JP Isaacs NW Cogdell RJ Jones MR Structural details of an interaction between cardiolipin and an integral membrane protein Proc Natl Acad Sci USA 1999 96 26 14706 14711 10.1073/pnas.96.26.14706 10611277 PMC24712

[B9] Mileykovskaya E Zhang M Dowhan W Cardiolipin in energy transducing membranes Biochem Mosc 2005 70 2 154 158 10.1007/s10541-005-0095-2 15807653

[B10] Romantsov T Helbig S Culham DE Gill C Stalker L Wood JM Cardiolipin promotes polar localization of osmosensory transporter ProP in Escherichia coli Mol Microbiol 2007 64 6 1455 1465 10.1111/j.1365-2958.2007.05727.x 17504273

[B11] Gold VA Robson A Bao H Romantsov T Duong F Collinson I The action of cardiolipin on the bacterial translocon Proc Natl Acad Sci USA 2010 107 22 10044 10049 10.1073/pnas.0914680107 20479269 PMC2890433

[B12] Corcelli A The cardiolipin analogues of Archaea Biochimica et Biophysica Acta-Biomembranes 2009 1788 10 2101 2106 10.1016/j.bbamem.2009.05.010 19464258

[B13] Daiyasu H Kuma K Yokoi T Morii H Koga Y Toh H A study of archaeal enzymes involved in polar lipid synthesis linking amino acid sequence information, genomic contexts and lipid composition Archaea 2005 1 6 399 410 10.1155/2005/452563 16243780 PMC2685579

[B14] Nowicki M Muller F Frentzen M Cardiolipin synthase of *Arabidopsis thalian* FEBS Lett 2005 579 10 2161 2165 10.1016/j.febslet.2005.03.007 15811335

[B15] Houtkooper RH Akbari H van Lenthe H Kulik W Wanders RJA Frentzen M Vaz FM Identification and characterization of human cardiolipin synthase FEBS Lett 2006 580 13 3059 3064 10.1016/j.febslet.2006.04.054 16678169

[B16] Schlame M Thematic review series: glycerolipids--cardiolipin synthesis for the assembly of bacterial and mitochondrial membranes J Lipid Res 2008 49 8 1607 1620 10.1194/jlr.R700018-JLR200 18077827 PMC2444000

[B17] Beranek A Rechberger G Knauer H Wolinski H Kohlwein SD Leber R Identification of a cardiolipin-specific phospholipase encoded by the gene CLD1 (YGR110W) in Yeast J Biol Chem 2009 284 17 11572 11578 19244244 10.1074/jbc.M805511200 PMC2670162

[B18] Malhotra A Edelman-Novemsky I Xu Y Plesken H Ma JP Schlame M Ren MD Role of calcium-independent phospholipase A(2) in the pathogenesis of Barth syndrome Proc Natl Acad Sci USA 2009 106 7 2337 2341 10.1073/pnas.0811224106 19164547 PMC2650157

[B19] Zachman DK Chicco AJ McCune SA Murphy RC Moore RL Sparagna GC The role of calcium-independent phospholipase A(2) in cardiolipin remodeling in the spontaneously hypertensive heart failure rat heart J Lipid Res 2010 51 3 525 534 10.1194/jlr.M000646 19741254 PMC2817582

[B20] Gu ZM Valianpour F Chen SL Vaz FM Hakkaart GA Wanders RJA Greenberg ML Aberrant cardiolipin metabolism in the yeast taz1 mutant: a model for Barth syndrome Mol Microbiol 2004 51 1 149 158 14651618 10.1046/j.1365-2958.2003.03802.x

[B21] Cao JS Liu YF Lockwood J Burn P Shi YG A novel cardiolipin-remodeling pathway revealed by a gene encoding an endoplasmic reticulum-associated acyl-CoA: lysocardiolipin acyltransferase (ALCAT1) in mouse J Biol Chem 2004 279 30 31727 31734 10.1074/jbc.M402930200 15152008

[B22] Kutschera U Niklas KJ Endosymbiosis, cell evolution, and speciation Theory Biosci 2005 124 1 1 24 10.1016/j.thbio.2005.04.001 17046345

[B23] de Andrade Rosa I Einicker-Lamas M Roney Bernardo R Previatto LM Mohana-Borges R Morgado-Diaz JA Benchimol M Cardiolipin in hydrogenosomes: evidence of symbiotic origin Eukaryot Cell 2006 5 4 784 787 10.1128/EC.5.4.784-787.2006 16607026 PMC1459669

[B24] Rosa ID Einicker-Lamas M Bernardo RR Benchimol M Cardiolipin, a lipid found in mitochondria, hydrogenosomes and bacteria was not detected in *Giardia lambli* Exp Parasitol 2008 120 3 215 220 10.1016/j.exppara.2008.07.009 18691575

[B25] Guschina IA Harris KM Maskrey B Goldberg B Lloyd D Harwood JL The microaerophilic flagellate, *Trichomonas vaginali*, contains unusual acyl lipids but no detectable cardiolipin J Eukaryot Microbiol 2009 56 1 52 57 10.1111/j.1550-7408.2008.00365.x 19335774

[B26] Benchimol M Hydrogenosomes under microscopy Tissue Cell 2009 41 3 151 168 10.1016/j.tice.2009.01.001 19297000

[B27] Gillin FD Reiner DS McCaffery JM Cell biology of the primitive eukaryote Giardia lamblia Annu Rev Microbiol 1996 50 679 705 10.1146/annurev.micro.50.1.679 8905095

[B28] Cavalier-Smith T Eukaryotes with no mitochondria Nature 1987 326 6111 332 333 10.1038/326332a0 3561476

[B29] Cavalier-Smith T Archaebacteria and Archezoa Nature 1989 339 6220 100 101 10.1038/339100a0 2497352

[B30] Katayama K Sakurai I Wada H Identification of an Arabidopsis thaliana gene for cardiolipin synthase located in mitochondria FEBS Lett 2004 577 1-2 193 198 10.1016/j.febslet.2004.10.009 15527784

[B31] Sandoval-Calderon M Geiger O Guan ZQ Barona-Gomez F Sohlenkamp C A eukaryote-like cardiolipin synthase is present in *Streptomyces coelicolo *and in most Actinobacteria J Biol Chem 2009 284 26 17383 17390 10.1074/jbc.M109.006072 19439403 PMC2719378

[B32] Tropp BE Cardiolipin synthase from Escherichia coli Biochim Biophys Acta 1997 1348 1-2 192 200 9370333 10.1016/s0005-2760(97)00100-8

[B33] Koprivnjak T Zhang D Ernst CM Peschel A Nauseef WM Weiss JP Characterization of Staphylococcus aureus cardiolipin synthases 1 and 2 and their contribution to accumulation of cardiolipin in stationary phase and within phagocytes J Bacteriol 2011 193 16 4134 4142 10.1128/JB.00288-11 21665977 PMC3147675

[B34] Tanaka H Minakami R Kanaya H Sumimoto H Catalytic residues of group VIB calcium-independent phospholipase A2 (iPLA2gamma) Biochem Biophys Res Commun 2004 320 4 1284 1290 10.1016/j.bbrc.2004.05.225 15249229

[B35] Embley TM Martin W Eukaryotic evolution, changes and challenges Nature 2006 440 7084 623 630 10.1038/nature04546 16572163

[B36] Esser C Martin W Dagan T The origin of mitochondria in light of a fluid prokaryotic chromosome model Biol Lett 2007 3 2 180 184 10.1098/rsbl.2006.0582 17251118 PMC2375920

[B37] Richards TA van der Giezen M Evolution of the Isd11-IscS complex reveals a single alpha-proteobacterial endosymbiosis for all eukaryotes Mol Biol Evol 2006 23 7 1341 1344 10.1093/molbev/msl001 16648156

[B38] Lykidis A Comparative genomics and evolution of eukaryotic phospholipid biosynthesis Prog Lipid Res 2007 46 3-4 171 199 10.1016/j.plipres.2007.03.003 17512056

[B39] Weeks G Herring FG The lipid composition and membrane fluidity of Dictyostelium discoideum plasma membranes at various stages during differentiation J Lipid Res 1980 21 6 681 686 6252271

[B40] Adosraku RK Smith JD Nicolaou A Gibbons WA Tetrahymena thermophila: analysis of phospholipids and phosphonolipids by high-field 1H-NMR Biochim Biophys Acta 1996 1299 2 167 174 8555261 10.1016/0005-2760(95)00181-6

[B41] Andrews D Nelson DL Biochemical studies of the excitable membrane of Paramecium tetraurelia. II. Phospholipids of ciliary and other membranes Biochim Biophys Acta 1979 550 2 174 187 10.1016/0005-2736(79)90205-0 758943

[B42] Soudant P Chu FL Marty Y Lipid class composition of the protozoan Perkinsus marinus, an oyster parasite, and its metabolism of a fluorescent phosphatidylcholine analog Lipids 2000 35 12 1387 1395 10.1007/s11745-000-0656-1 11202001

[B43] Oliveira MM Timm SL Costa SC Lipid composition of Trypanosoma cruzi Comp Biochem Physiol B 1977 58 2 195 199 10.1016/0305-0491(77)90109-2 400952

[B44] Zillig W Comparative biochemistry of Archaea and Bacteria Curr Opin Genet Dev 1991 1 4 544 551 10.1016/S0959-437X(05)80206-0 1822288

[B45] Cavalier-Smith T The phagotrophic origin of eukaryotes and phylogenetic classification of protozoa Int J Syst Evol Microbiol 2002 52 297 354 11931142 10.1099/00207713-52-2-297

[B46] Hartman H Fedorov A The origin of the eukaryotic cell: a genomic investigation Proc Natl Acad Sci USA 2002 99 3 1420 1425 10.1073/pnas.032658599 11805300 PMC122206

[B47] Horiike T Hamada K Kanaya S Shinozawa T Origin of eukaryotic cell nuclei by symbiosis of Archaea in Bacteria is revealed by homology-hit analysis Nat Cell Biol 2001 3 2 210 214 10.1038/35055129 11175755

[B48] Lake JA Rivera MC Was the nucleus the first endosymbiont? Proc Natl Acad Sci USA 1994 91 8 2880 2881 10.1073/pnas.91.8.2880 8159671 PMC43475

[B49] El Alaoui H Bata J Bauchart D Dore JC Vivares CP Lipids of three microsporidian species and multivariate analysis of the host-parasite relationship J Parasitol 2001 87 3 554 559 11426718 10.1645/0022-3395(2001)087[0554:LOTMSA]2.0.CO;2

[B50] Haines TH A new look at Cardiolipin Biochim Biophys Acta 2009 1788 10 1997 2002 10.1016/j.bbamem.2009.09.008 19801076

[B51] Jakovcic S Getz GS Rabinowitz M Jakob H Swift H Cardiolipin content of wild type and mutant yeasts in relation to mitochondrial function and development J Cell Biol 1971 48 3 490 502 10.1083/jcb.48.3.490 4322761 PMC2108117

[B52] Pereto J Lopez-Garcia P Moreira D Ancestral lipid biosynthesis and early membrane evolution Trends Biochem Sci 2004 29 9 469 477 10.1016/j.tibs.2004.07.002 15337120

[B53] Jenkins CM Han X Mancuso DJ Gross RW Identification of calcium-independent phospholipase A2 (iPLA2) beta, and not iPLA2gamma, as the mediator of arginine vasopressin-induced arachidonic acid release in A-10 smooth muscle cells J Biol Chem 2002 277 36 32807 32814 10.1074/jbc.M202568200 12089145

[B54] Serfontein J Nisbet RE Howe CJ de Vries PJ Evolution of the TSC1/TSC2-TOR signaling pathway Sci Signal 2010 3 128 ra49 10.1126/scisignal.2000803 20587805

[B55] Stechmann A Cavalier-Smith T Rooting the eukaryote tree by using a derived gene fusion Science 2002 297 5578 89 91 10.1126/science.1071196 12098695

[B56] Burki F Shalchian-Tabrizi K Pawlowski J Phylogenomics reveals a new 'megagroup' including most photosynthetic eukaryotes Biol Lett 2008 4 4 366 369 10.1098/rsbl.2008.0224 18522922 PMC2610160

[B57] Hampl V Hug L Leigh JW Dacks JB Lang BF Simpson AG Roger AJ Phylogenomic analyses support the monophyly of Excavata and resolve relationships among eukaryotic "supergroups" Proc Natl Acad Sci USA 2009 106 10 3859 3864 10.1073/pnas.0807880106 19237557 PMC2656170

[B58] Roger AJ Reconstructing early events in eukaryotic evolution Am Nat 1999 154 S146 S163 10.1086/303290 10527924

[B59] Roger AJ Clark CG Doolittle WF A possible mitochondrial gene in the early-branching amitochondriate protist *Trichomonas vaginali* Proc Natl Acad Sci USA 1996 93 25 14618 14622 10.1073/pnas.93.25.14618 8962102 PMC26183

[B60] Tovar J Fischer A Clark CG The mitosome, a novel organelle related to mitochondria in the amitochondrial parasite Entamoeba histolytica Mol Microbiol 1999 32 5 1013 1021 10.1046/j.1365-2958.1999.01414.x 10361303

[B61] Tovar J Leon-Avila G Sanchez LB Sutak R Tachezy J van der Giezen M Hernandez M Muller M Lucocq JM Mitochondrial remnant organelles of Giardia function in ironsulphur protein maturation Nature 2003 426 6963 172 176 10.1038/nature01945 14614504

[B62] Hrdy I Hirt RP Dolezal P Bardonova L Foster PG Tachezy J Embley TM Trichomonas hydrogenosomes contain the NADH dehydrogenase module of mitochondrial complex I Nature 2004 432 7017 618 622 10.1038/nature03149 15577909

[B63] Edgar RC MUSCLE: a multiple sequence alignment method with reduced time and space complexity BMC Bioinformatics 2004 5 113 10.1186/1471-2105-5-113 15318951 PMC517706

[B64] Price MN Dehal PS Arkin AP FastTree 2-approximately maximum-likelihood trees for large alignments PLoS One 2010 5 3 e9490 10.1371/journal.pone.0009490 20224823 PMC2835736

[B65] Ronquist F Huelsenbeck JP MrBayes 3: Bayesian phylogenetic inference under mixed models Bioinformatics 2003 19 12 1572 1574 10.1093/bioinformatics/btg180 12912839

[B66] Whelan S Goldman N A general empirical model of protein evolution derived from multiple protein families using a maximum-likelihood approach Mol Biol Evol 2001 18 5 691 699 10.1093/oxfordjournals.molbev.a003851 11319253

[B67] Stamatakis A Hoover P Rougemont J A rapid bootstrap algorithm for the RAxML Web servers Syst Biol 2008 57 5 758 771 10.1080/10635150802429642 18853362

[B68] Shimodaira H Hasegawa M CONSEL: for assessing the confidence of phylogenetic tree selection Bioinformatics 2001 17 12 1246 1247 10.1093/bioinformatics/17.12.1246 11751242

