# Supplementary material for: The evolution of cardiolipin biosynthesis and maturation pathways and its implications for the evolution of eukaryotes
Source: BMC Evol Biol. 2012 Mar 13;12:32. doi: 10.1186/1471-2148-12-32 (PMC3378450; doi:10.1186/1471-2148-12-32)
Supplement: Additional file 1 — Identified homologs involved in CLS synthesis and maturation pathways in eukaryotes. [file 1471-2148-12-32-S1.DOC]

**Additional file S2.** Identified homologs involved in CLS synthesis and maturation pathways in eukaryotes.

| **Organism** | **CLS-cap** | **CLS-pld** | **CLD** | **iPLA_2_** | | **ALCAT** | **TAZ** |
| --- | --- | --- | --- | --- | --- | --- | --- |
|  |  |  |  | **beta** | **gamma** |  |  |
| *Homo sapien* | NP_061968.1  NP_001120930.1 | - | NP_071343.2  NP_057090.2 | NP_001004426.1  NP_003551.2 | NP_056538.1 | NP_872357.2  NP_001002257.1 | NP_000107.1  NP_851829.1  NP_851830.1  NP_851828.1 |
| *Mus musculus* | NP_079922.2  NP_001019556.1 | - | NP_598837.1  NP_080455.1 | NP_058611.1 | NP_080440.2 | NP_001074540.1 | NP_852657.1  NP_001167018.1 |
| *Xenopus laevis* | NP_001090462.1 | - | NP_001086565.1 | NP_001086854.1 | NP_001086854.1 | NP_001135517.1 | NP_001079572.1 |
| *Gallus gallus* | XP_426115.2 | - | NP_001006365.1 | NP_001124210.1 | NP_001124209.1 | NP_001026210.1 | BX933903.1(EST) |
| *Danio rerio* | NP_998096.1 | - | XP_694855.2 NP_001017613.1 | NP_998262.1 | XP_001918731.2 | NP_998435.1 | NP_001001814.1 |
| *Drosophila melanogaster* | NP_651418.1  NP_733116.1  NP_733117.1 | - | NP_724609.1  NP_610326.1 | NP_729565.2  NP_648366.2 | - | - | NP_477432.3  NP_725227.2  NP_725226.2 |
| *Caenorhabditis elegans* | NP_001022546.1  NP_001022547.1 | - | NP_492685.2  NP_504297.1  NP_504299.2 | NP_491201.2  NP_501821.2  NP_509011.1  NP_501497.1  NP_872170.1  NP_508000.1  NP_001123029.1  NP_509647.1 | NP_500969.1 | NP_504643.1  NP_504644.1  NP_505971.2 | NP_502202.1 |
| *Hydra magnipapillata* | XP_002154402.1  XP_002164157.1 | - | XP_002156782.1  XP_002156802.1  XP_002156760.1 | XP_002162202.1  XP_002162270.1  XP_002164080.1  XP_002162284.1 | - | XP_002158894.1 | Contig38390 |
| *Strongylocentrotus purpuratus* | XP_792193.2  XP_001189252.1 | - | XP_780777.2  XP_001197390.1 | XP_781913.2  XP_001186249.1 | XP_782958.1  XP_001183199.1 | XP_795667.2  XP_782691.1 | XP_001183204.1 |
| *Schistosoma mansoni* | XP_002574764.1 | - | XP_002575733.1 | - | XP_002573501.1 | XP_002580449.1 | XP_002577764.1 |
| *Ciona intestinalis* | XP_002131552.1 | - | XP_002121163.1 | XP_002119484.1 | XP_002126910.1 | XP_002119716.1  XP_002129599.1 | XP_002120143.1 |
| *Monosiga brevicollis* | XP_001749288.1 | - | XP_001750136.1 | - | - | XP_001747405.1 | XP_001748742.1 |
| *Saccharomyces cerevisiae* | NP_010139.1 | - | NP_011625.1 | - | - | NP_010301.1 | NP_015466.1 |
| *Schizosaccharomyces pombe* | NP_593238.1 | - | NP_594100.1 | - | - | NP_595192.1 | - |
| *Aspergillus* *fumigatus* | XP_747632.1 | - | XP_750139.1 | XP_755122.1 | - | XP_754044.1 | XP_755729.1 |
| *Ustilago maydis 521* | XP_756829.1 | - | XP_762408.1 | - | - | XP_762206.1 | XP_756829.1 |
| *Cryptococcus neoformans* | XP_568890.1 | - | XP_570770.1  XP_775332.1 | - | - | XP_568878.1  XP_568877.1 | XP_777272.1  XP_568890.1 |
| *Arabidopsis thaliana* | NP_567273.1 | - | NP_194147.2 | - | NP_176378.4 | NP_188515.1  NP_565098.1  NP_001078184.1 | NP_177990.1  NP_974228.1  NP_566254.1 |
| *Oryza sativa* | NP_001044486.1 | - | NP_001063697.1 | - | NP_001175227.1 | NP_001044448.1 | NP_001045296.2  NP_001054183.1 |
| *Chlamydomonas reinhardtii* | XP_001699073.1 | - | XP_001700328.1 | - | XP_001702734.1 | - | XP_001702919.1 |
| *Ostreococcus lucimarinus* | XP_001422530.1 | - | XP_001420632.1 | - | XP_001418186.1  XP_001417740.1 | - | XP_001421134.1 |
| *Ostreococcus tauri* | XP_003082955.1 | - | CAL57254.1 | - | CAL53414.1  CAL53973.1 | - | CAL56577.1 |
| *Micromonas sp. RCC299* | XP_002505710.1  XP_002507590.1 | - | XP_002506430.1 | XP_002502975.1 | XP_002501836.1  XP_002502482.1 | XP_002501997.1 | XP_002502967.1 |
| *Cyanidioschyzon merolae* | CMER|CMN196C | - | CMER|CML191C | - | CMER|CMR500C  CMER|CMT312C | - | CMP142C |
| *Galdieria sulphuraria* (EST) | HET_11H01 | - | - | - | - | - | HET_2D4 |
| *Dictyostelium discoideum* | - | XP_638476.2 | - | XP_639007.1  XP_639008.1  XP_639009.2  XP_629835.1  XP_647314.1  XP_636272.1  XP_642421.1  XP_636632.1  XP_635843.1  XP_629480.1  XP_629694.1 | - | XP_646339.1  XP_636688.1 | XP_629919.1 |
| *Dictyostelium purpureum* | - | DPU0052781|DPU_G0052780 | - | DPU0073051|DPU_G0073050  DPU0059843|DPU_G0059842  DPU0056029|DPU_G0056028  DPU0061861|DPU_G0061860  DPU0055293|DPU_G0055292  DPU0056027|DPU_G0056026  DPU0056959|DPU_G0056958 | - | DPU0060745|DPU_G0060744 | DPU0052989|DPU_G0052988 |
| *Leishmania braziliensis* | - | AM494957(LbrM20_V2.1600) | XP_001565089.1 | - | - | XP_001563860.1 | - |
| *Leishmania infantum* | - | XP_001468797.1 | XP_001465693.1 | - | - | XP_001464760.1 | - |
| *Leishmania major* | - | XP_001686308.1 | XP_001683337.1 | - | - | XP_001682349.1 | - |
| *Trypanosoma brucei* | - | XP_844406.1 | XP_847044.1 | - | - | XP_844902.1 | - |
| *Trypanosoma cruzi* | - | XP_821784.1  XP_806232.1 | AAP30860.1 | - | - | XP_808202.1 XP_814400.1 | - |
| *Tetrahymena thermophila* | - | XP_001030185.1 | XP_001033485.1  XP_001027360.1  XP_001030577.1  XP_001011412.1  XP_001011413.1  XP_001019416.1  XP_001017356.1 | - | - | - | - |
| *Paramecium tetraurelia* | - | XP_001432739.1 | XP_001450800.1  XP_001459425.1 | - | - | - | - |
| *Perkinsus marinus* | - | XP_002784805.1 | - | - | XP_002775091.1 | XP_002768928.1 | - |
| *Naegleria gruberi* | - | XP_002677818.1 | - | - | XP_002674773.1  XP_002670499.1  XP_002672956.1  XP_002673476.1 | - | XP_002680872.1 |
| *Plasmodium knowlesi* | - | XP_002261580.1 | - | - | XP_002261312.1 | - | - |
| *Plasmodium vivax* | - | XP_001616116.1 | - | - | XP_001616364.1 | - | - |
| *Plasmodium faciparum* | - | XP_966084.1 | - | - | XP_001350291.1 | - | - |
| *Plasmodium chabaudi* | - | XP_746201.1 | - | - | XP_672403.1 | - | - |
| *Plasmodium yoelli yoelii* | - | XP_727656.1 | - | - | - | - | - |
| *Cryptosporidium parvum* | - | XP_626851.1 | - | - | - | - | - |
| *Cryptosporidium hominis* | - | XP_666798.1 | - | - | - | - | - |
| *Cryptosporidium muris* | - | XP_002141525.1 | - | - | - | - | - |
| *Toxoplasma gondii* | - | XP_002364240.1 | - | - | XP_002368028.1 | - | - |
| *Babesia bovis T2Bo* | - | XP_001609724.1 | - | - | XP_001611186.1 | - | - |
| *Theileria parva* | - | XP_763964.1 | - | - | XP_762910.1 | - | - |
| *Theileria annulata* | - | XP_952999.1 | - | - | XP_955468.1 | - | - |
| *Antonospora locustae* | - | - | - | - | - | - | - |
| *Encephalitozoon cuniculi* | - | - | - | - | - | - | - |
| *Enterocytozoon bieneusi* | - | - | - | - | - | - | - |
| *Encephalitozoon intestinalis* | - | - | - | - | - | - | - |
| *Entamoeba histolytica* | - | - | - | - | - | - | - |
| *Entamoeba dispar* | - | - | - | - | - | - | - |
| *Entamoeba invadens* | - | - | - | - | - | - | - |
| *Trichomonas vaginalis* | - | - | - | - | - | - | - |
| *Giardia lamblia* | - | - | - | - | - | - | - |
| *Blastocystis hominis* | - | - | - | - | - | CBK23989.2  CBK24473.2 | CBK22384.2  CBK20032.2 |
| *Thalassiosira pseudonana* CCMP1335 | Thaps3|260941 | - | Thaps3|264297  Thaps3|269487 | - | Thaps3|23984 | - | Thaps3|24714| |
| *Phaeodactylum tricornutum* CCAP 1055/1 | Chr24_400817-401665  Chr24_8494-9340 | - | Phatr2|54974 | - | Phatr2|46193 | Phatr2|45551 | - |
| *Ectocarpus siliculosus* | Esi0244_0030 | - | Esi0176_0002  Esi0040_0068 | - | Esi0036_0001  Esi0179_0062 | Esi0491_0009  Esi0055_0099  Esi0161 0029 | Esi0047_0048  Esi0104_0041 |
| *Pythium ultimum* BR144 | PYU1_T006543 | - | PYU1_T002291  PYU1_T002292  PYU1_T010216  PYU1_T010217 | - | - | PYU1_T012544  PYU1_T010731  PYU1_T012835 | PYU1_T010480 |
| *Phytophthora sojae* | Ps_140356T0 | - | Ps_157051T0 | Ps_157051  Ps_155786T0 | Ps_155786 | - | - | Ps_141240T0  Ps_141831T0  Ps_157022T0 | - |
| *Phytophthora ramorum* | PITG_17041T0 | PITG_17041 | - | Pr_95977T0 | Pr_95977  Pr_93863T0 | Pr_93863 | - | - | Pr_79555T0 | Pr_79555  Pr_73463T0 | Pr_73463 | Pr_74800T0 | Pr_74800 |
| *Phytophthora infestans* | Pr_43567T0 | Pr_43567 | - | PITG_03525T0 | PITG_03525  PITG_12144T0 | PITG_12144  PITG_12145T0 | PITG_12145 | - | - | PITG_10763T0 | PITG_10763  PITG_06638T0 | PITG_06638  PITG_02075T0 | PITG_02075 | PITG_06716T0 | PITG_06716 |
| *Saprolegnia parasitica*  CBS 223.65 | SPRG_02155T0 | SPRG_02155 | - | SPRG_02569T0 | SPRG_02569 | - | - | SPRG_00156T0 | SPRG_00156  SPRG_02994T0 | SPRG_02994  SPRG_04915T0 | SPRG_04915  SPRG_16315T0 | SPRG_16315 | SPRG_06619T0 | SPRG_06619  SPRG_16301T0 | SPRG_16301 |
