# Supplementary material for: The evolution of cardiolipin biosynthesis and maturation pathways and its implications for the evolution of eukaryotes
Source: BMC Evol Biol. 2012 Mar 13;12:32. doi: 10.1186/1471-2148-12-32 (PMC3378450; doi:10.1186/1471-2148-12-32)
Supplement: Additional file 2 — Figure S1. The alignment of CLS_cap of eukaryotes (part). Conserved six membrane-binding regions are designated as I-VI and conserved amino acid residues among CAP family are boxed. Amino acid positions are numbered relative to the Monosiga brevicollis ortholog. # below the alignment indicates the amino acid residues that are specific for CL synthases. [file 1471-2148-12-32-S2.PDF]

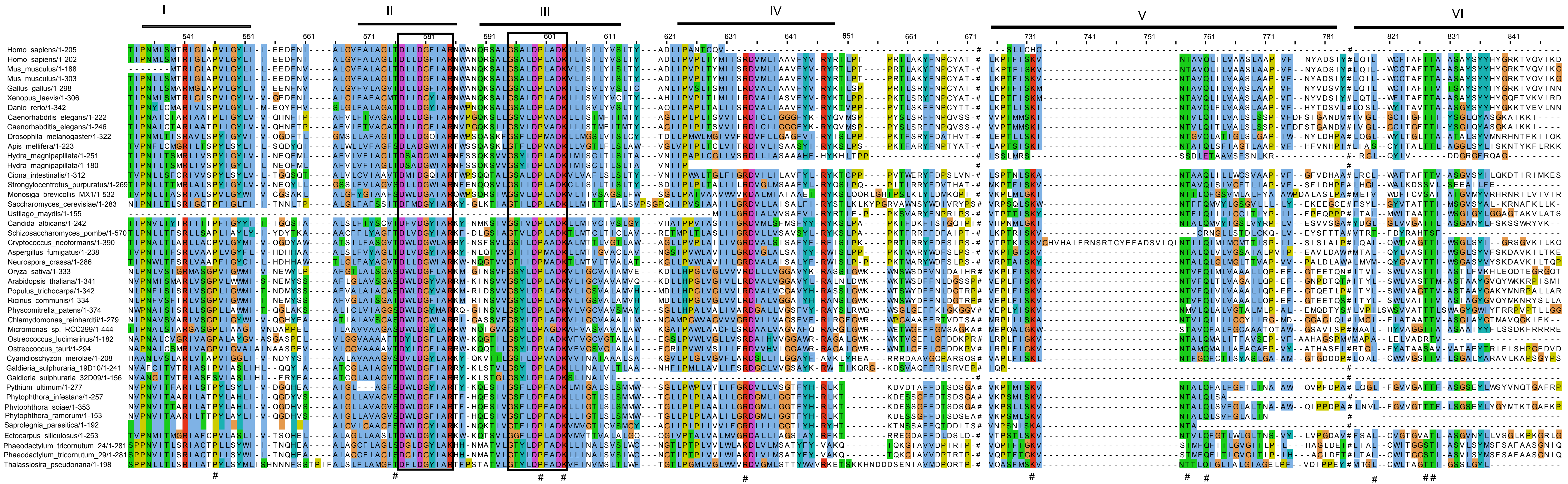
I
II
III
IV
V
VI
Homo_sapiens/1-205
Homo_sapiens/1-202
Mus_musculus/1-188
Mus_musculus/1-303
Gallus_gallus/1-298
Xenopus_laevis/1-306
Danio_rerio/1-342
Caenorhabditis_elegans/1-222
Caenorhabditis_elegans/1-246
Drosophila_melanogaster/1-322
Apis_mellifera/1-223
Hydra_magnipapillata/1-251
Hydra_magnipapillata/1-180
Ciona_intestinalis/1-312
Strongylocentrotus_purpuratus/1-269
Monosiga_brevicollis_MX1/1-532
Saccharomyces_cerevisiae/1-283
Ustilago_maydis/1-155
Candida_albicans/1-242
Schizosaccharomyces_pombe/1-570
Cryptococcus_neoformans/1-390
Aspergillus_fumigatus/1-238
Neurospora_crassa/1-286
Oryza_sativa/1-333
Arabidopsis_thaliana/1-341
Populus_trichocarpa/1-342
Ricinus_communis/1-334
Physcomitrella_patens/1-374
Chlamydomonas_reinhardtii/1-279
Micromonas_sp._RCC299/1-444
Ostreococcus_lucimarinus/1-182
Ostreococcus_tauri/1-294
Cyanidioschyzon_merolae/1-208
Galdieria_sulphuraria_19D10/1-241
Galdieria_sulphuraria_32D09/1-156
Pythium_ultimum/1-277
Phytophthora_infestans/1-257
Phytophthora_sojae/1-353
Phytophthora_ramorum/1-153
Saprolegnia_parasitica/1-192
Ectocarpus_siliculosus/1-253
Phaeodactylum_tricornutum_24/1-281
Phaeodactylum_tricornutum_29/1-281
Thalassiosira_pseudonana/1-198
