# Supplementary material for: The evolution of cardiolipin biosynthesis and maturation pathways and its implications for the evolution of eukaryotes
Source: BMC Evol Biol. 2012 Mar 13;12:32. doi: 10.1186/1471-2148-12-32 (PMC3378450; doi:10.1186/1471-2148-12-32)
Supplement: Additional file 3 — Figure S2. The identified conserved motifs (the boxed regions) of CLS_pld from mitochondriate protists. Amino acid positions are numbered relative to the Plasmodium knowlesi ortholog (gi: 221058144). [file 1471-2148-12-32-S3.PDF]

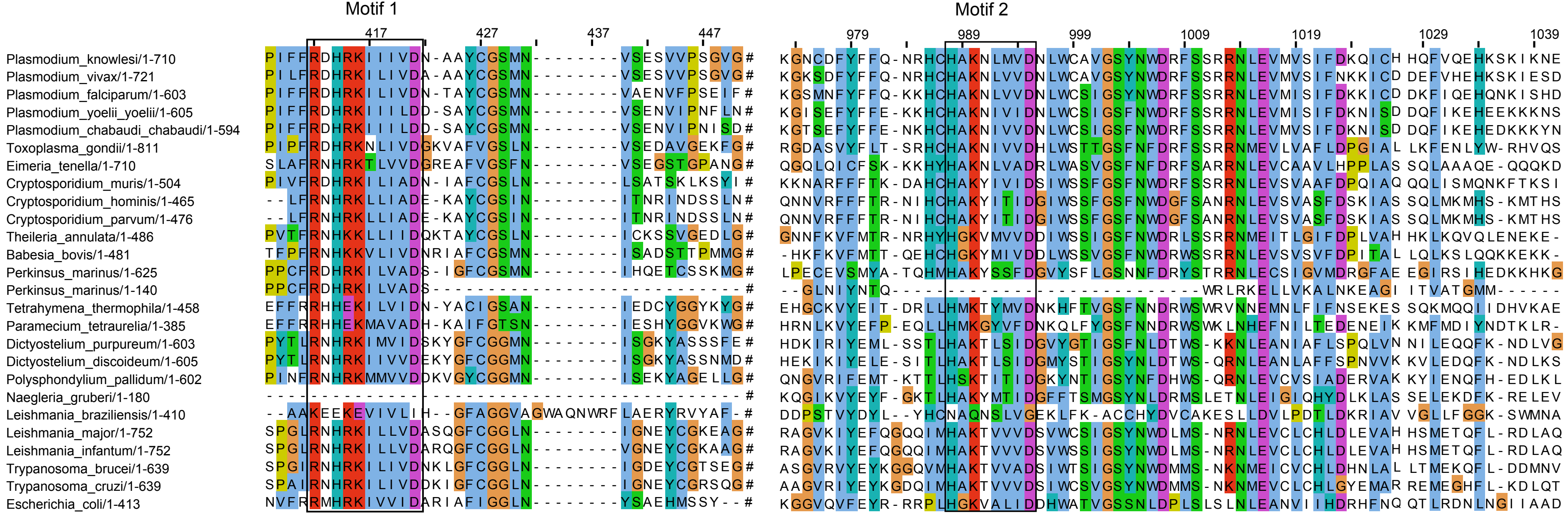
Motif 1
Motif 2
417
427
437
447
979
989
999
1009
1019
1029
1039
Plasmodium_knowlesi/1-710
Plasmodium_vivax/1-721
Plasmodium_falciparum/1-603
Plasmodium_yoelii_yoelii/1-605
Plasmodium_chabaudi_chabaudi/1-594
Toxoplasma_gondii/1-811
Eimeria_tenella/1-710
Cryptosporidium_muris/1-504
Cryptosporidium_hominis/1-465
Cryptosporidium_parvum/1-476
Theileria_annulata/1-486
Babesia_bovis/1-481
Perkinsus_marinus/1-625
Perkinsus_marinus/1-140
Tetrahymena_thermophila/1-458
Paramecium_tetraurelia/1-385
Dictyostelium_purpureum/1-603
Dictyostelium_discoideum/1-605
Polysphondylium_pallidum/1-602
Naegleria_gruberi/1-180
Leishmania_braziliensis/1-410
Leishmania_major/1-752
Leishmania_infantum/1-752
Trypanosoma_brucei/1-639
Trypanosoma_cruzi/1-639
Escherichia_coli/1-413
