# Supplementary figures and images for: The evolution of cardiolipin biosynthesis and maturation pathways and its implications for the evolution of eukaryotes
Source: BMC Evol Biol. 2012 Mar 13;12:32. doi: 10.1186/1471-2148-12-32 (PMC3378450; doi:10.1186/1471-2148-12-32)

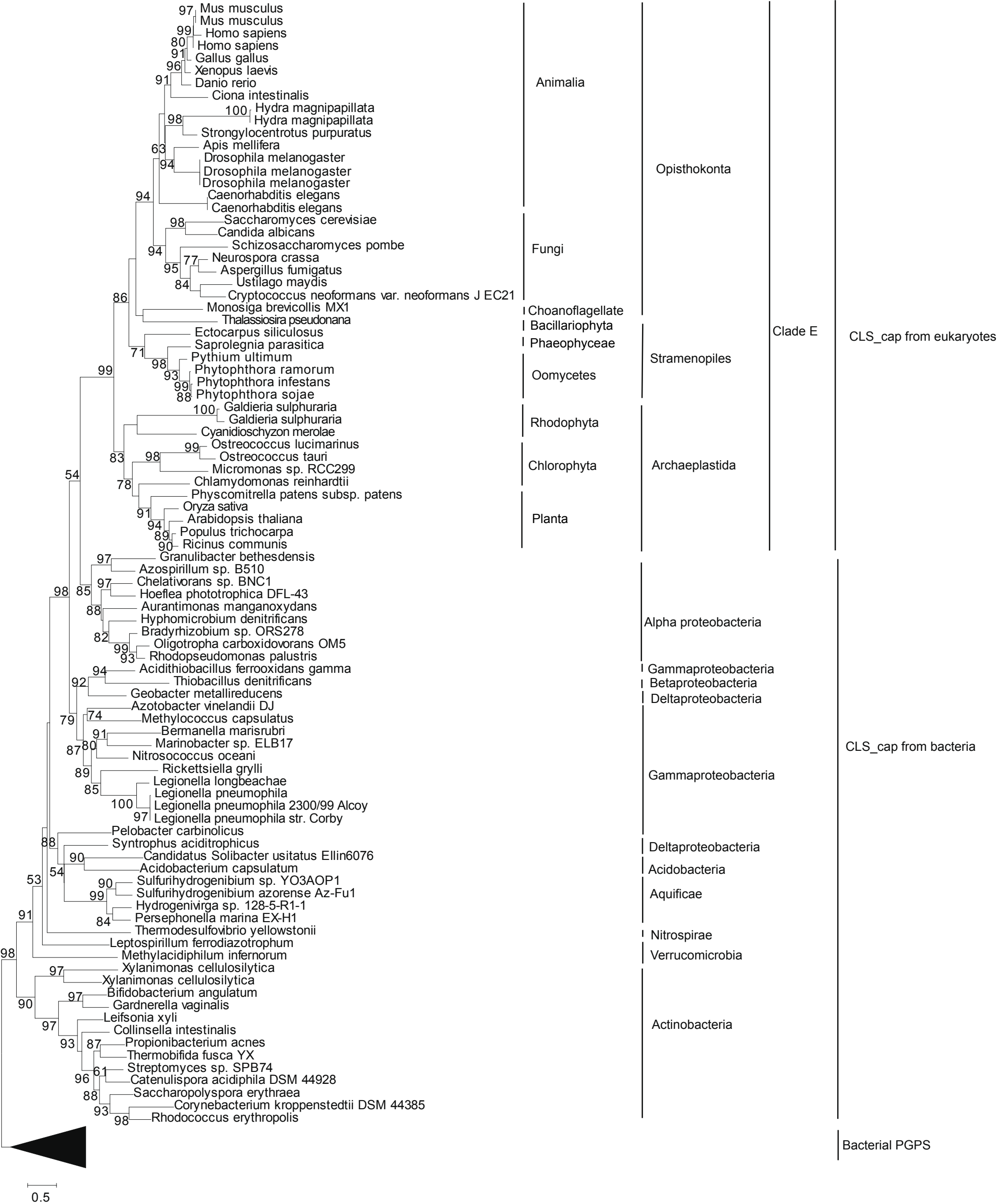

Supplement: Additional file 6 — Figure S5. The ML phylogenetic tree of all the CLS_cap from eukaryotes and bacteria, and PGPS homologs of bacteria, which is corresponding to the Bayesian tree of Figure 2. [file 1471-2148-12-32-S6.PDF]

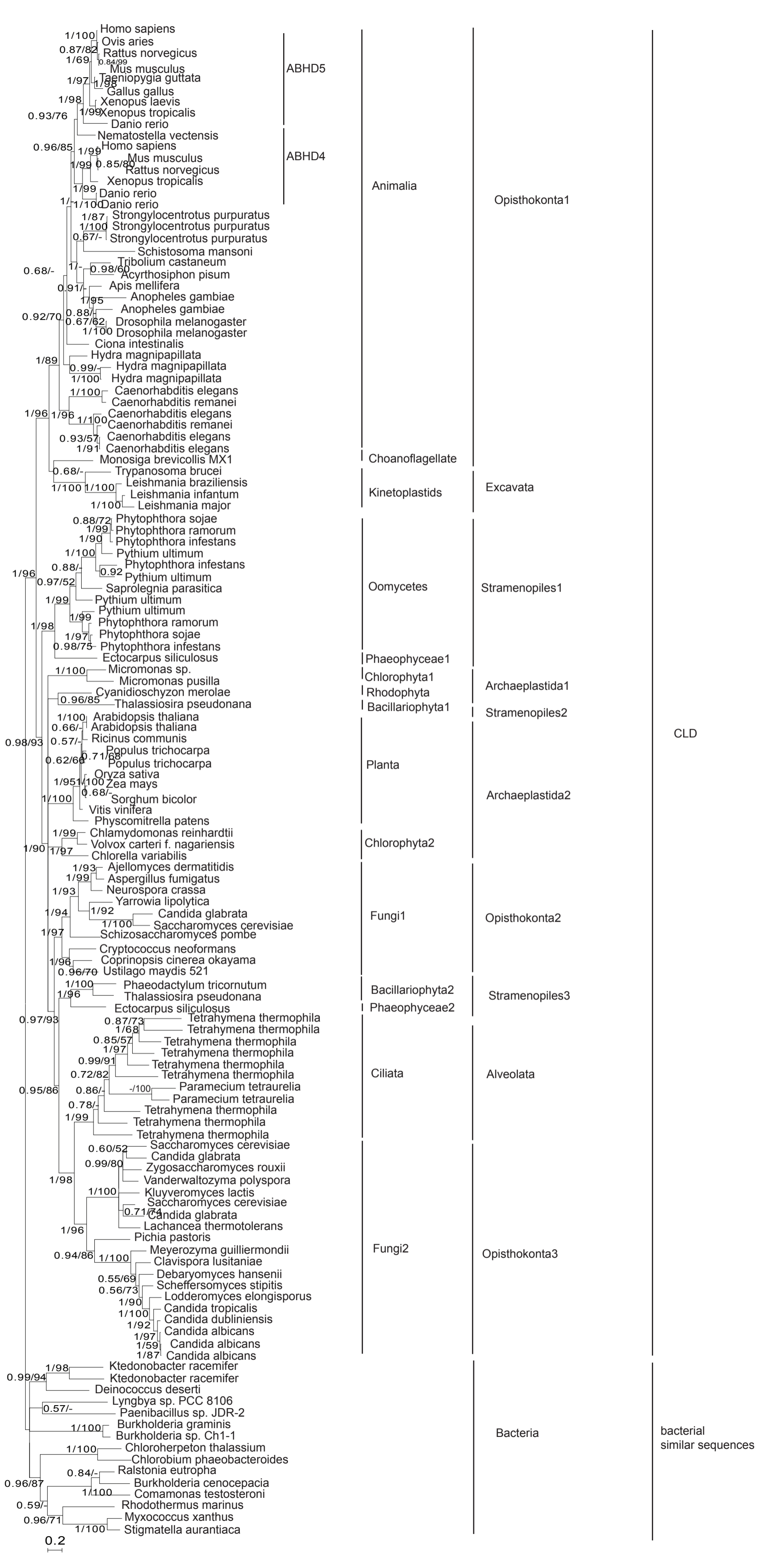

Supplement: Additional file 7 — Figure S6. Phylogeny of eukaryotic homologs of CLD and bacterial similar sequences. The tree was constructed by using MrBayes 3.1.2, and is illustrated using the same conventions as Figure 1. The monophyly constraint of Fungi (Fungi1+Fungi2) passed the AU test, suggesting they might be obtained through lineage-specific gene duplication. [file 1471-2148-12-32-S7.PDF]

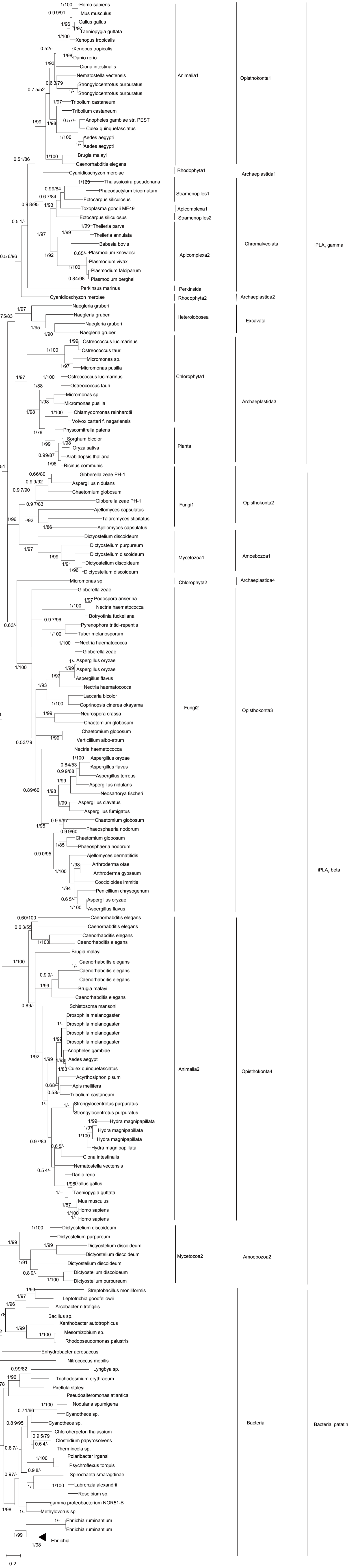

Supplement: Additional file 8 — Figure S7. Phylogeny of iPLA2 and related bacterial similar sequences. The tree was constructed by using MrBayes 3.1.2, and is illustrated using the same conventions as Figure 1. The rejection of monophyly hypothesis of Animalia (Animalia1+Animalia2) by AU test (0.048) argues that iPLA2 beta and gamma diverged in the ancestor of Animalia though it's hard to determine the time. [file 1471-2148-12-32-S8.PDF]

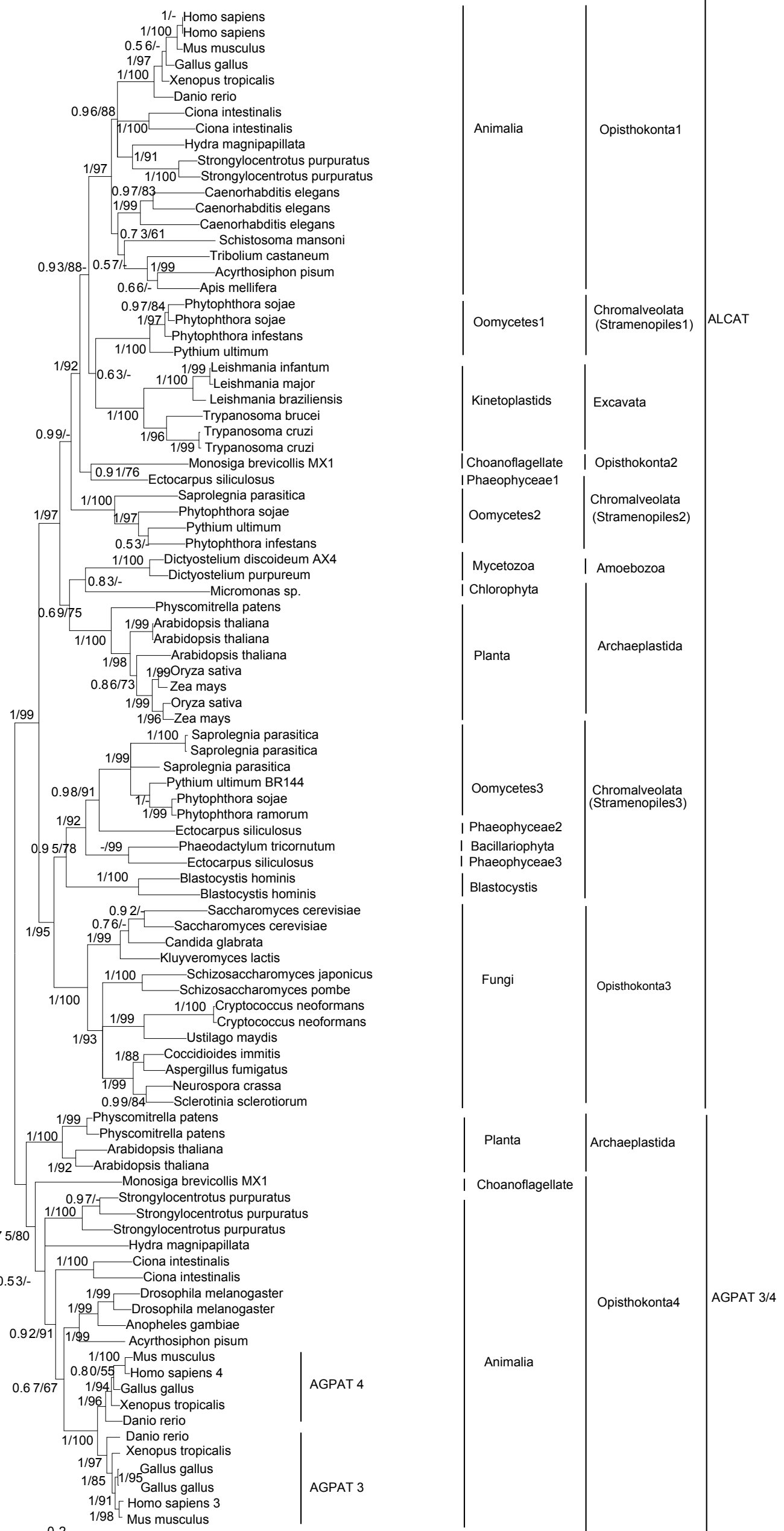

Supplement: Additional file 9 — Figure S8. Phylogeny of ALCAT and AGPAT 3/4. The tree was constructed by using MrBayes 3.1.2, and is illustrated using the same conventions as Figure 1. AGPAT 3/4 were rooted as outgroup based on our preliminary analyses. The tree is illustrated using the same conventions as in Figure 1. Alternative trees constraining all Stramenopiles as monophyly were rejected, suggesting gene duplication occurred in the ancestor of Stramenopiles. [file 1471-2148-12-32-S9.PDF]

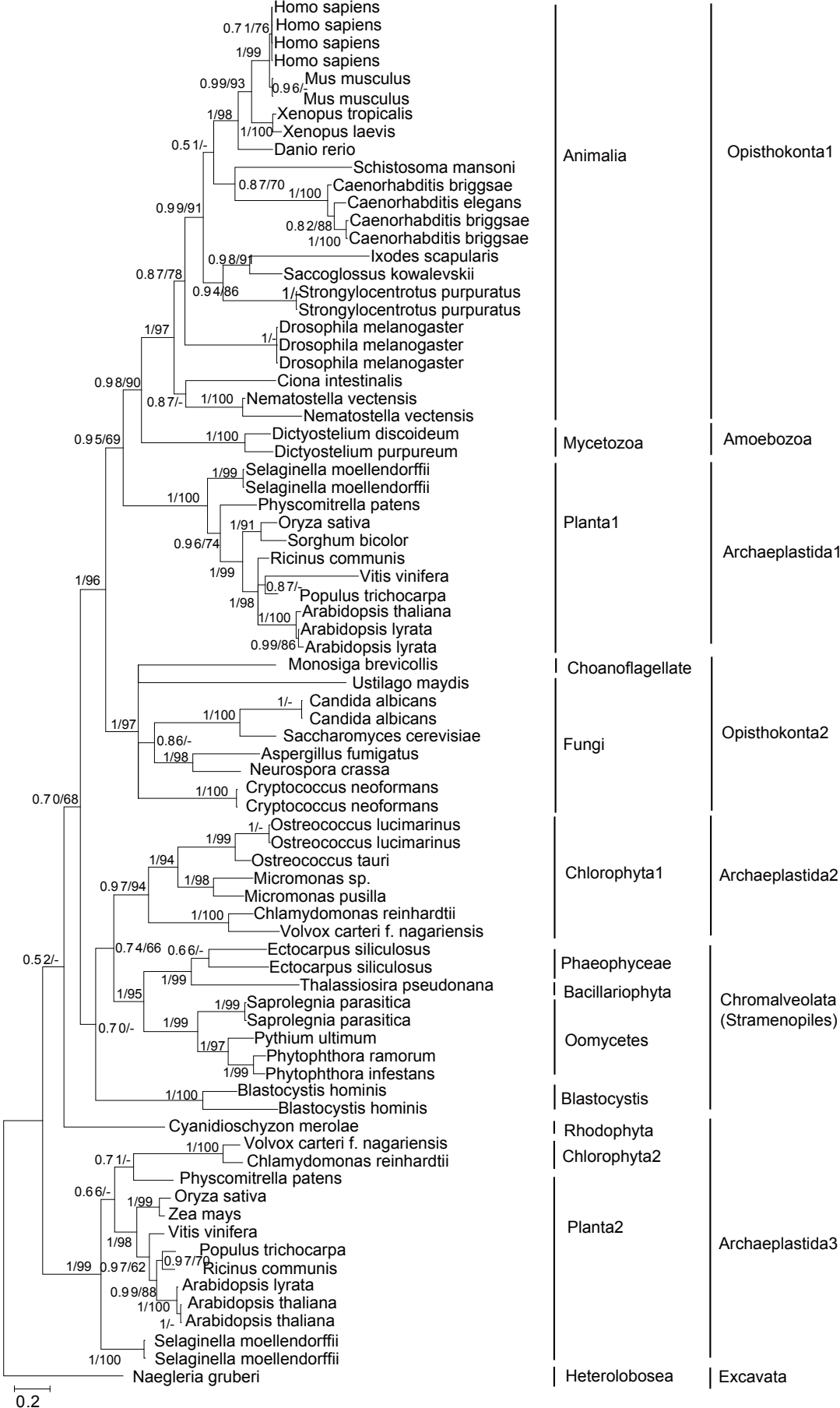

Supplement: Additional file 10 — Figure S9. Phylogeny of the TAZ and bacterial similar sequences. The tree was constructed by using MrBayes 3.1.2, and is illustrated using the same conventions as Figure 1. Hypothetical trees constraining all Archaeplastids as monophyly were rejected, suggesting gene duplication occurred in the ancestor of Archaeplstids. [file 1471-2148-12-32-S10.PDF]
