# Supplementary material for: The evolution of cardiolipin biosynthesis and maturation pathways and its implications for the evolution of eukaryotes
Source: BMC Evol Biol. 2012 Mar 13;12:32. doi: 10.1186/1471-2148-12-32 (PMC3378450; doi:10.1186/1471-2148-12-32)
Supplement: Additional file 11 — Additional file S11. Comparision between Bayesian tree and alternative topologies [file 1471-2148-12-32-S11.DOC]

**Additional file S12**, Comparision between Bayesian tree and alternative topologies.

CLD

| Tree | Description | AU Probability |
| --- | --- | --- |
| 1 | Bayesian tree | 0.602 |
| 2 | (Animal+Choanoflagellate) | 0.619 |
| 3 | (Fungi1+Fungi2) | 0.086 |
| 4 | (Animal+Choanoflagellate+Fungi1) | 0.006** |
| 5 | (Animal+Choanoflagellate+Fungi2) | 0.003** |
| 6 | (Animal+Choanoflagellate+Fungi1+Fungi2) | 0.024* |
| 7 | (Plants+Chlorophyta1) | 0.333 |
| 8 | (Plants+Chlorophyta1+Rhodophyta) | 0.198 |
| 9 | (Plants+Chlorophyta2) | 0.635 |
| 10 | (Plants+Chlorophyta1+Rhodophyta+Chlorophyta2) | 0.614 |
| 11 | (Stramenopiles1+ Stramenopiles2) | 0.302 |
| 12 | (Stramenopiles1+ Stramenopiles2+ Stramenopiles3) | 0.217 |
| 13 | (Stramenopiles3+Alveolata) | 0.078 |
| 14 | (Stramenopiles1+ Stramenopiles2+ Stramenopiles3+Alveolata) | 0.029* |

Note—Significantly rejected trees are marked with * when *P* < 0.05 and with ** when *P* < 0.01.

iPLA_2_

| Tree | Description | AU Probability |
| --- | --- | --- |
| 1 | Bayesian tree | 0.541 |
| 2 | (Animal1+Animal2) | 0.048* |
| 3 | (Fungi1+Fungi2 ) | 0.366 |
| 4 | (Plant+ Chlorophyta1+ Rhodophyta1+ Rhodophyta2) | 0.046* |
| 5 | (Plant+ Chlorophyta1+ Rhodophyta1+ Rhodophyta2+Chlorophyta2) | 0.016* |
| 6 | (Animal2+Fungi2+ Chlorophyta2+ Mycetozoa2) | 0.448 |
| 7 | (Mycetozoa1+Mycetozoa2) | 0.348 |
| 8 | (Stramenopiles) | 0.607 |
| 9 | (Apicomplexa) | 0.380 |
| 10 | (Stramenopiles+Apicomplexa+Dinoflagellate) | 0.657 |
| 11 | (Animal2+ Fungi1+Fungi2+ Chlorophyta2+ Mycetozoa1+Mycetozoa2) | 0.587 |
| 12 | (Animal2+ Fungi1+Fungi2+ Plant+ Chlorophyta1+Chlorophyta2+ Mycetozoa1+Mycetozoa2) | 0.166 |
| 13 | (Animal2+ Fungi1+Fungi2+ Plant+ Chlorophyta1+ Rhodophyta1+ Rhodophyta2+Chlorophyta2+ Mycetozoa1+Mycetozoa2) | 0.004** |
| 14 | (Rhodophyta1+Rhodophyta2) | 0.203 |
| 15 | (Plant+ Chlorophyta1+ Rhodophyta1) | 0.063 |
| 16 | (Plant+ Chlorophyta1+ Rhodophyta2) | 0.365 |
| 17 | (Plant+ Chlorophyta1+ Chlorophyta2) | 0.474 |

Note—Significantly rejected trees are marked with * when *P* < 0.05 and with ** when *P* < 0.01.

ALCAT

| Tree | Description | AU Probability |
| --- | --- | --- |
| 1 | Bayesian tree | 0.582 |
| 2 | (Animal+Choanoflagellate) | 0.346 |
| 3 | (Oomycetes1+brown algae1 ) | 0.118 |
| 4 | (Oomycetes1+ Oomycetes2) | 0.117 |
| 5 | (Oomycetes1+ Oomycetes2+ Oomycetes3) | 4e-004** |
| 6 | (Oomycetes2+ brown algae1) | 0.392 |
| 7 | (Oomycetes1+Oomycetes2+ brown algae1) | 0.200 |
| 8 | Archaeplastida1(Plants+ Chlorophyta) | 0.363 |
| 9 | (Animal+Fungi+Choanoflagellate) | 0.001** |
| 10 | (brown algae1+brown algae2) | 5e-005** |
| 11 | (brown algae1+brown algae2+brown algae3) | 4e-005** |
| 12 | S3 | 0.782 |
| 13 | S1S2S3 | 2e-006** |

Note—Significantly rejected trees are marked with * when *P* < 0.05 and with ** when *P* < 0.01.

TAZ

| Tree | Description | AU Probability |
| --- | --- | --- |
| 1 | Bayesian tree | 0.793 |
| 2 | (Plants1+Chlorophyta1) | 5e-004** |
| 3 | (Plants1+Chlorophyta1+Rhodophyta) | 0.006** |
| 4 | (Plants1+Chlorophyta1+Rhodophyta+Plants2+Chlorophyta2) | 0.003** |
| 5 | (Rhodophyta+Plants2+Chlorophyta2) | 0.282 |
| 6 | (Animals+Choanoflagellate) | 0.031* |
| 7 | (Animals+Choanoflagellate+Fungi) | 0.173 |
| 8 | （Stramenopiles） | 0.530 |

Note—Significantly rejected trees are marked with * when *P* < 0.05 and with ** when *P* < 0.01.
