# Supplementary material for: The evolution of cardiolipin biosynthesis and maturation pathways and its implications for the evolution of eukaryotes
Source: BMC Evol Biol. 2012 Mar 13;12:32. doi: 10.1186/1471-2148-12-32 (PMC3378450; doi:10.1186/1471-2148-12-32)
Supplement: Additional file 12 — Additional file S12. The download sites of eukaryotic genomes or EST database included in the analyses [file 1471-2148-12-32-S12.DOC]

**Additional file S1** The download sites of eukaryotic genomes or EST database included in the analyses.

| Organisms | Download sites |
| --- | --- |
| *Homo sapiens* | NCBI |
| *Gallus gallus* | NCBI |
| *Danio rerio* | NCBI |
| *Drosophila melanogaster* | NCBI |
| *Caenorhabditis elegans* | NCBI |
| *Hydra magnipapillata* | NCBI |
| *Strongylocentrotus purpuratus* | NCBI |
| *Schistosoma mansoni* | NCBI |
| *Ciona intestinalis* | NCBI |
| *Monosiga brevicollis* | *http://genome.jgi-psf.org/Monbr1/Monbr1.home.html* |
| *Saccharomyces cerevisiae* | NCBI |
| *Schizosaccharomyces pombe* | NCBI |
| *Aspergillus* *fumigatus* | NCBI |
| *Ustilago maydis* 521 | NCBI |
| *Cryptococcus neoformans* | NCBI |
| *Antonospora locustae* | <http://forest.mbl.edu/cgi-bin/site/antonospora01?page=download> |
| *Encephalitozoon cuniculi* | NCBI |
| *Enterocytozoon bieneusi* | <http://microsporidiadb.org/common/downloads/release-1.2/> |
| *Encephalitozoon intestinalis* | <http://microsporidiadb.org/common/downloads/release-1.2/> |
| *Dictyostelium discoideum* | NCBI |
| *Dictyostelium purpureum* | [*http://dictybase.org/db/cgi-bin/dictyBase/download/blast_databases.pl*](http://dictybase.org/db/cgi-bin/dictyBase/download/blast_databases.pl) |
| *Entamoeba histolytica* | NCBI |
| *E. dispar* | http://amoebadb.org/common/downloads/ |
| *E. invadens* | http://amoebadb.org/common/downloads/ |
| *Arabidopsis thaliana* | NCBI |
| *Oryza sativa* | NCBI |
| *Chlamydomonas reinhardtii* | *http://genome.jgi-psf.org/chlamy/chlamy.home.html* |
| *Ostreococcus l**ucimarinus* | NCBI |
| *Ostreococcus tauri* | NCBI |
| *Micromonas sp.* RCC299 | NCBI |
| *Galdieria sulphuraria* (EST) | <http://genomics.msu.edu/cgi-bin/galdieria/blast.cgi> |
| *Cyanidioschyzon merolae* | http://merolae.biol.s.u-tokyo.ac.jp/download/ |
| *Tetrahymena thermophila* | NCBI |
| *Paramecium tetraurelia* | http://paramecium.cgm.cnrs-gif.fr/parameciumDB |
| *Perkinsus marinus* | NCBI |
| *Thalassiosira pseudonana* CCMP1335 | http://genome.jgi-psf.org/Thaps3/Thaps3.download.ftp.html |
| *Phaeodactylum tricornutum* CCAP 1055/1 | http://genome.jgi-psf.org/Phatr2/Phatr2.download.ftp.html |
| *Phytophthora sojae* | http://genome.jgi-psf.org/Physo2/Physo2.home.html |
| *Phytophthora ramorum* | http://genome.jgi-psf.org/Physo2/Physo2.home.html |
| *Pythium ultimum* BR144 | http://pythium.plantbiology.msu.edu/download.html |
| *Phytophthora infestans* | http://www.broadinstitute.org/annotation/genome/phytophthora_infestans/Downloads.html;jsessionid=F9EE33E8E49BBC60989A87F0FC370283.route990 |
| *Saprolegnia parasitica*  CBS 223.65 | http://www.broadinstitute.org/annotation/genome/Saprolegnia_parasitica/MultiDownloads.html |
| *Ectocarpus siliculosus* | https://bioinformatics.psb.ugent.be/gdb/ectocarpus/ |
| *Plasmodium knowlesi/ vivax/ faciparum/chabaudi/yoelli yoelii* | *EupathDB* |
| *Cryptosporidium parvum/hominis/ muris* | *EupathDB* |
| *Toxoplasma gondii* | *EupathDB* |
| *Babesia bovis* | *EupathDB* |
| *Theileria annulata/parva* | *EupathDB* |
| *Leishmania braziliensis/ infantum/major* | *EupathDB* |
| *Trypanosoma brucei/cruzi* | *EupathDB* |
| *Blastocystis hominis* | NCBI |
| *Trichomonas vaginalis* | *EupathDB* |
| *Giardia lamblia* | *EupathDB* |
